# Pangenome Analysis of *Clostridium scindens*: A Collection of Diverse Bile Acid- and Steroid-Metabolizing Commensal Gut Bacterial Strains

**DOI:** 10.3390/microorganisms13040857

**Published:** 2025-04-09

**Authors:** Kelly Y. Olivos-Caicedo, Francelys V. Fernandez-Materan, Steven L. Daniel, Karthik Anantharaman, Jason M. Ridlon, João M. P. Alves

**Affiliations:** 1 Department of Parasitology, Institute of Biomedical Sciences, University of São Paulo, São Paulo 05508-000, Brazil; kellyolivos@usp.br; 2 Microbiome Metabolic Engineering Theme, Carl R. Woese Institute for Genomic Biology, Urbana, IL 61801, USA; fvf3@illinois.edu; 3 Department of Animal Sciences, University of Illinois at Urbana-Champaign, Urbana, IL 61801, USA; cfsld@illinois.edu; 4 Department of Biological Sciences, Eastern Illinois University, Charleston, IL 61920, USA; 5 Department of Bacteriology, University of Wisconsin-Madison, Madison, WI 53706, USA; karthik@bact.wisc.edu; 6 Department of Data Science and AI, Indian Institute of Technology Madras, Chennai 600036, India; 7 Division of Nutritional Sciences, University of Illinois at Urbana-Champaign, Urbana, IL 61801, USA; 8 Cancer Center at Illinois, University of Illinois at Urbana-Champaign, Urbana, IL 61801, USA; 9 Department of Microbiology and Immunology, Virginia Commonwealth University School of Medicine, Richmond, VA 23298, USA

**Keywords:** *Clostridium scindens*, bile acids, deoxycholic acid, 7α-dehydroxylation, core genome, pangenome

## Abstract

*Clostridium scindens* is a commensal gut bacterium capable of forming the secondary bile acids as well as converting glucocorticoids to androgens. Historically, only two strains, *C. scindens* ATCC 35704 and *C. scindens* VPI 12708, have been characterized to any significant extent. The formation of secondary bile acids is important in the etiology of cancers of the GI tract and in the prevention of *Clostridioides difficile* infection. We determined the presence and absence of bile acid inducible (*bai*) and steroid-17,20-desmolase (*des*) genes among *C. scindens* strains and the features of the pangenome of 34 cultured strains of *C. scindens* and a set of 200 metagenome-assembled genomes (MAGs) to understand the variability among strains. The results indicate that the *C. scindens* cultivars have an open pangenome with 12,720 orthologous gene groups and a core genome with 1630 gene families, in addition to 7051 and 4039 gene families in the accessory and unique (i.e., strain-exclusive) genomes, respectively. The pangenome profile including the MAGs also proved to be open. Our analyses reveal that *C. scindens* strains are distributed into two clades, indicating the possible onset of *C. scindens* separation into two species, as suggested by gene content, phylogenomic, and average nucleotide identity (ANI) analyses. This study provides insight into the structure and function of the *C. scindens* pangenome, offering a genetic foundation of significance for many aspects of research on the intestinal microbiota and bile acid metabolism.

## 1. Introduction

The emulsification of dietary lipids in the aqueous milieu of the vertebrate small bowel represents a fundamental problem that was solved through the evolution of a complex pathway in the liver that converts cholesterol into detergents known as primary bile acids [1]. The term “secondary bile acid” was coined in 1960, denoting microbial conversion products of host primary bile acids produced in the liver [2]. That year, the same research group proposed a two-step mechanism for bile acid 7α-dehydroxylation that we have referred to as the Samuelsson–Bergstrӧm model [2]. Yet, the cultivation and preservation of anaerobic bacteria capable of bile acid 7α-dehydroxylation were enduring problems that slowed progress in the field [2]. It was not until the early 1980s that strains of *Clostridium scindens* (i.e., VPI 12708 and ATCC 35704) were isolated and characterized so that the Samuelsson–Bergström model could be adequately tested [3,4,5,6].

**Figure 1:**
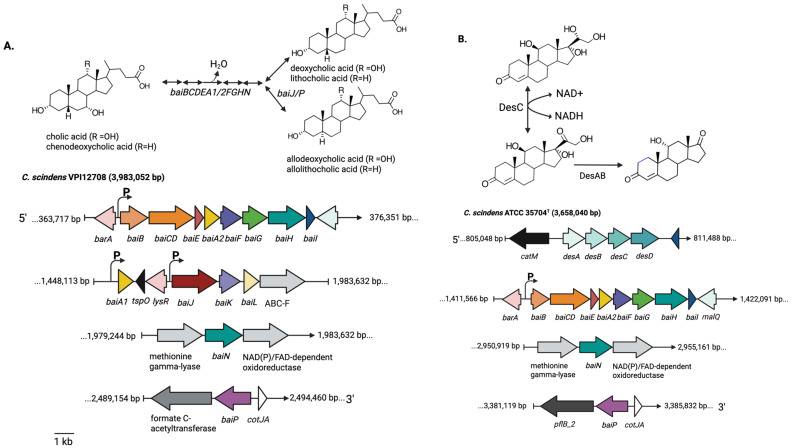
Steroid and bile acid biotransformation pathways: *C. scindens* VPI 12708 pathway (**A**) and *C. scindens* ATCC 35704 pathway (**B**).

In 1991, a new, more complex model of bile acid 7α-dehydroxylation was proposed, which we have recently termed the Hylemon–Bjӧrkhem (HB) pathway, that uniquely explains the formation of allo-secondary bile acids (Figure 1) [2]. A bile-acid-inducible (*bai*) regulon in *C. scindens* VPI 12708 was cloned during the 1990s, and studies spanning the subsequent three decades have uncovered enzymes catalyzing each step of the HB pathway [7] (Figure 1). This biochemical pathway involved in 7α-dehydroxylation of bile acids is encoded by the *bai* operon, which is restricted to a phylogenetic group of bacterial species belonging to the *Clostridium* clusters IV (*Ruminococcaceae*), XI (*Peptostreptococcaceae*), and XIVa (*Lachnospiraceae*), of which the most extensively studied species in *Lachnospiraceae* are *C. scindens* and *Clostridium hylemonae* [2,8,9,10,11], and most recently *Faecalicatena contorta* [12].

There is much current interest in the role of *bai* genes and in the formation of hydrophobic secondary bile acids such as deoxycholic acid (DCA) and lithocholic acid (LCA) in gastrointestinal (GI) diseases [7]. For many decades, the enrichment of DCA and LCA associated with Western Diets high in animal protein and saturated fat was linked with increased risk for colorectal cancer (CRC) [13]. Diet exchange studies demonstrate that high-animal-protein and high-fat diets drive the elevation of bile-acid-metabolizing genes and functional activities such as increased bile salt hydrolase (*bsh*) and bile-acid-inducible (*bai*) genes [14,15]. Indeed, there is compelling evidence for the co-carcinogenic role of hydrophobic secondary bile acids in the GI tract [16]. Meta-analysis of metagenomic studies reveals an enrichment of *bai* genes in CRC [17]. The antimicrobial nature of secondary bile acids also results in lower gut microbial diversity [18], which is also associated with development of CRC [19]. A recent study provided compelling evidence that disruption of *baiH* in *F. contorta* reduces dextran-sodium-sulfate-induced colitis in mice [12]. *C. scindens* also appears to exacerbate bile acid diarrhea, at least in mice, through alteration in liver production of primary bile acids that inhibit ileal FGF15 production through decreased FXR activation [20].

Current thinking about secondary bile acids has expanded in recent years as new evidence accumulates regarding the importance of maintaining moderate levels of DCA and LCA [13,21]. *Clostridioides difficile* infection [22] and inflammatory bowel disease [23] are associated with gut dysbiosis and low levels of fecal secondary bile acids and enrichment in primary conjugated bile acids. *C. scindens* remains one of limited taxa in the phylum *Bacillota* capable of forming DCA and LCA in the vertebrate GI tract [7]. Studies with complex consortia of gut bacteria both in vitro and in vivo confirm that, despite their low abundance in the gut microbiome, the expression of functional *bai* genes by bile acid 7α-dehydroxylating bacteria is a necessary condition for generating DCA and LCA from primary bile acids [12,24]. It has been suggested that the re-establishment of DCA levels in feces by the introduction of *C. scindens* may be therapeutic in the treatment and prevention of *C. difficile* (re)infection [22,25]. This points to the potential for *C. scindens* or the *bai* pathway to have therapeutic benefit in certain acute or even chronic conditions in the GI tract [26,27]. By contrast, controlling the output of primary bile acids and maintaining moderate levels of DCA and LCA through diets low in animal protein and fat and high in vegetables and fiber may be beneficial in other clinical contexts such as CRC [19].

Yet, our understanding of the biology and diversity of *C. scindens* is very limited to date, with much of the in vitro and in vivo work focusing on *C. scindens* ATCC 35704 [10,24,28], the type strain, and *C. scindens* VPI 12708 [2], the first isolate of this species shown to be capable of 7α-dehydroxylation. Are there strain(s) of *C. scindens* with therapeutic potential, and are there some strains that should be avoided? While assumed, it has not been determined whether the *bai* pathway genes are found in most (or all) strains of this species, and the extent of genomic diversity between strains of *C. scindens* is currently unknown. In selection of potentially therapeutic strains, it will be important to have baseline information relating to the genomic diversity of a gut bacterial species [29].

Here, we present a current comprehensive genomic study of *C. scindens* strains from both cultured isolates and metagenome-assembled genomes (MAGs). We use *C. hylemonae* as an outgroup and point of comparison for the *C. scindens* strains since *C. hylemonae* is, among well-characterized isolates, the 7α-dehydroxylating bacterium species that is most closely related to *C. scindens* known to date. Our major findings are as follows: (1) All strains identified as *C. scindens* possess the *bai* regulon but vary with respect to individual *bai* genes; there is also significant variation with respect to the *des* pathway genes. (2) Isolates with >97% gene identity in the full-length 16S rRNA sequence separate into two groups, which present ~4–5% difference in genomic sequence based on average nucleotide identity (ANI), suggesting that potentially two separate microbial species are present, or are in the process of speciation. (3) While the core genome is closed, the pangenome remains open, indicating that additional strain diversity exists.

An open pangenome can be extrapolated into a sympatric lifestyle and the ability to gain new species-specific genes, which could be related to functions such as virulence, metabolism, and information storage, among others [30]. An open pangenome also suggests the possibility of adding new gene sets, along with new strain-specific genes (singletons). Furthermore, the persistence of singletons might represent the ability to acquire new virulence traits, a threat to human health. Likewise, an open pangenome indicates that it is necessary to study more genomes, from a greater variety of environments and geographic locations, in order to define the entire genomic content of this (or these) species.

## 2. Materials and Methods

### 2.1. Genomic Sequences

The sequenced genomes of 34 strains of *C. scindens* were analyzed (Table 1). These included: (1) 8 complete genomes (number of contigs = 1) and 2 incomplete genomes (number of contigs = 2 and 5) that were recently published by our group [31,32]; (2) 7 complete genomes (number of contigs = 1) obtained from the public GenBank database at the National Center for Biotechnology Information (NCBI), and (3) 17 incomplete genomes (number of contigs ranging from 21 to 797) obtained from NCBI. In Table 1, additional characteristics are described, such as accession number, host, and geographic origin, among others.

The strains used in the present study were isolated from North America, Europe, and Asia, most from human fecal samples of healthy adults, some from unhealthy adults, and two strains isolated from pig and mouse fecal samples (Table 1). Two hundred *C. scindens* genomes were assembled from metagenomic data (MAGs) obtained from the intestinal metagenome of human fecal samples [33,34,35].

### 2.2. Genome Annotation

Sequencing data quality was assessed with the FastQC tool version 0.11.8 [36], and the correction and de novo assembling of genomes were performed with Unicycler version 0.5.0 [37], Flye version 2.9 [38], and Canu version 2.2 [39], resulting in either complete (i.e., circularized) or incomplete (i.e., fragmented into a few contigs) genome assemblies. Evaluation of genome assembly completeness was performed with BUSCO (Benchmarking Universal Single-Copy Ortholog) tool version 5.3.1 [40], and the *Clostridium* database of 247 orthologous genes. In addition, completeness and contamination were assessed with CheckM tool version 1.2.2 [41], using a lineage-specific workflow. Functional annotation of the 34 genomes and MAGs of *C. scindens* was performed with Prokka version 1.14 [42], using default parameters values. Prokka performs the prediction of protein-coding genes (CDS), tRNAs, and rRNAs on bacterial, archaeal, and viral genomes, generating individual whole-genome annotation files in a GFF format for each strain, later used in diverse downstream analysis, such as the pangenome analysis detailed below.

### 2.3. Metagenome-Assembled Genomes (MAGs) of C. scindens from Public Metagenomes

Genomes from human gut microbiome datasets were downloaded from the following nine different sources: 32,277 genomes from Zeng et al. 2022 [33], 1200 genomes from Wilkinson et al. 2020 [43], 120 genomes classified as *C. scindens* from Almeida et al. 2020 [35], 1381 genomes from Tamburini et al. 2021 [44], 154,723 genomes from Pasolli et al. 2019 [34], 4997 genomes from Carter et al. 2023 [45], 2914 genomes from Lemos et al. 2022 [46], 4497 genomes from Gounot et al. 2022 [47], and 31 genomes from NCBI. The GTDB-Tk (version 2.1.1) classify workflow (classify_wf) was run on all 202,140 genomes, which resulted in the identification of 224 *C. scindens* genomes, including 200 MAGs.

To generate a list of non-redundant *C. scindens* genomes, we used the program dRep version 3.4.0 [48] on the group of 224 MAGs. The process of dRep includes identifying genomes that are essentially the same and removing all genomes identified as the same except for the best genome that represents that cluster of identical genomes. In this case, the requirement was to have an ANI of 99% for two genomes to be considered the same.

### 2.4. Determination of the Pangenome of 34 C. scindens Strains

The *C. scindens* pangenome and gene content variation of the 34 genomes and 200 MAGs were determined based on the annotation file generated by Prokka and the Roary tool version 3.13.0 [49]. Roary defines clusters of homologous proteins between genomes, thus identifying orthologous genes. To evaluate the MAGs’ pangenome, percentage values of genome completeness were considered for including a MAG in the analyses, testing completeness values from 50% to 100%, increasing by 5% at a time. A 95% identity and 99% definition were used as minimum standard criteria for BLASTP and the core genome in this study, allowing Roary to classify genes present in ≥99% of the genomes as core genes, genes present in at least two strains as shell or accessory genes, and genes specific to each strain as cloud or unique genes.

In general, the Roary pipeline iteratively filtered and pre-clustered proteins with CD-Hit version 4.8.1 [50] then performed an all-against-all comparison using BLASTP version 2.9.0+; subsequently, sequences were clustered with the Markov cluster algorithm (MCL) [51], and finally the CD-HIT pre-clustering results were merged together with the MCL results. Moreover, the FastTree tool version 2.1.11 [52] and a Roary script (roary_plots.py) were used to visualize a presence–absence matrix of core and accessory genes shared between genomes to determine the variation between the sets of strains. Additionally, the Roary binary matrix file for presence–absence of orthologous genes among all strains was selected to estimate the size of the pangenome and core genome using the PanGP version 1.0.1 program [53] with the “totally random sampling” algorithm. Heaps Law’s alpha parameter (to estimate whether the pangenome is closed or open) and core genome predicted size were also estimated using the micropan R package, version 2.1 [54].

### 2.5. Distance Analysis of Strains

ANI analysis was performed with the PyANI tool version 0.3.0 [55] with the “-m ANIb” option to indicate genome alignment using the ANIb method with BLAST and to study the variation between the nine newly sequenced genomes and the genomes obtained from NCBI, including *C. hylemonae* species as an outgroup. The suggested threshold percentage of ANI for species identification is greater than or equal to 95% [56], and that is the value we employed in our analyses in this work.

First, a multiple sequence alignment was performed with Muscle version 3.8 [57]. Subsequently, to create the distance matrix, the Distmat tool (EMBOSS version 6.6.0) was used, with a *C. hylemonae* strain included as an outgroup. The distances were generated using the “Uncorrected” setting for multiple substitution correction. Finally, a table was created with the generated matrix, to be represented by a heat map.

### 2.6. Functional Annotation: Prediction of COG and KEGG Groups

Functional annotations were assigned to proteins using the Clusters of Orthologous Genes (COG) database from the eggNOG-mapper v.2 online tool run against eggNOG’s version 5 database [58] (http://eggnog5.embl.de accessed on 29 January 2024).

The metabolic pathways were studied with the Kyoto Encyclopedia of Genes and Genomes (KEGG) database through the online tool KAAS v.2.1 (KEGG Automatic Annotation Server) [59], accessed on 3 January 2025. Orthologous gene families have been organized into classes and subclasses. Finally, the percentage frequencies of the COG and KEGG categories were calculated for the set of core genes, accessory genes, and unique genes.

### 2.7. Identification of bai and des Genes in C. scindens Genomes

To identify the presence of genes from the *bai* operon (bile-acid-inducible) and genes from the *desABC* operon (cortisol-inducible) in the 34 genomes and 200 MAGs of *C. scindens*, a similarity analysis was performed with the BLASTP 2.9.0+ tool [60], using *bai* and *des* genes from *C. scindens* ATCC 35704 and *C. scindens* VPI 12708 (Supplementary Table S1) as queries and the proteome database of the analyzed strains as a database, with a maximum allowed e-value set at 1E-20. Amino acid sequences with a similarity greater than 90% were considered as best matches. The sequences analyzed belong to the genes *baiA_2*, *baiB*, *baiCD*, *baiE*, *baiF*, *baiG*, *baiH*, *baiI*, *baiJ*, *baiK*, *baiN*, and *baiP*, and to the genes *desA*, *desB*, and *desC* obtained from *C. scindens* ATCC 35704 or *C. scindens* VPI 12708.

Custom HMMs were also generated for the Bai and DesA, DesB, and DesC proteins by using these experimentally verified sequences. Briefly, in order to create the protein alignments needed to generate HMM profiles, Muscle (5.1.linux64) was used with default parameters to align all amino acid sequences for each of the proteins followed by the hmmbuild function of the HMMER (version 3.3.2) package to generate the HMM profiles. Trusted HMM cutoffs were generated for each of the proteins based on the maximum F-scores based on searches with orthologous proteins. The 224 identified *C. scindens* genomes had their amino acid sequences predicted using Prodigal (version 2.6.3). The generated HMM profiles were queried against the amino acid sequences for the 224 genomes using hmmsearch of the HMMER package with the flag—cut_tc.

### 2.8. Phylogenomic Analysis

The Orthofinder v2.5.4 program [61] was used to analyze the proteomes of 34 cultured *C. scindens* strains and *C. hylemonae*. All protein sequences from orthogroups of interest were aligned with the Muscle program version 3.8. Gblocks version 0.91b [62] was used to remove ambiguously aligned regions, as these regions decrease the quality of phylogenetic inference. Default parameters were used, except for keeping columns where gaps occurred in up to half of the sequences in the alignment.

After eliminating ambiguous positions, alignments smaller than 50% of the original size were discarded. Finally, FASconCaT v1.04 [63] was used to concatenate all resulting alignments and create a supermatrix for phylogenetic inference analysis. The tree was generated using the maximum likelihood method with RAxML version 8.2.12 [64] and was drawn and edited manually with the online tool iTOL, accessed on 29 January 2025 (https://itol.embl.de/) and Inkscape version 1.3.

## 3. Results

### 3.1. Assembly and Genomic Characteristics of C. *scindens* Strains

Comparative analysis of the recently sequenced *C. scindens* strains and the genomes available at NCBI showed strain variation with respect to genome size. The genome lengths are between 3.2 and 4.6 Mbp, belonging to strains MSK.1.26 and DFI.1.130, respectively, and presenting 3140 to 4177 coding sequences (CDS). Percentage GC composition showed little variation, ranging from 45.5 to 47.8%, with strains BL389WT3D and VE202-05 having the lowest and highest values, respectively. Genomic characteristics of the 34 *C. scindens* strains, such as the number of contigs and RNA types, are shown in Table 2.

The CheckM tool was used to assess the completeness of the genome assembly of the 34 *C. scindens* strains using the *Lachnospiraceae* (UID1256) dataset (as determined automatically by the program) with 333 reference markers. All strains have a high quality, with almost complete sets of single-copy orthologous genes (between one and three markers missing) and completeness values above 98%, except strain VE202-05, which showed a greater amount of missing genes, 15, and therefore a completeness of 94.13% (Supplementary Table S2). Assembly completeness of the MAGs was also assessed by the CheckM tool, giving maximum and minimum values of 99.42% and 50.78%, respectively (Supplementary Tables S3 and S4).

### 3.2. Pangenome of the 34 Cultured Strains of C. *scindens*

**Figure 2:**
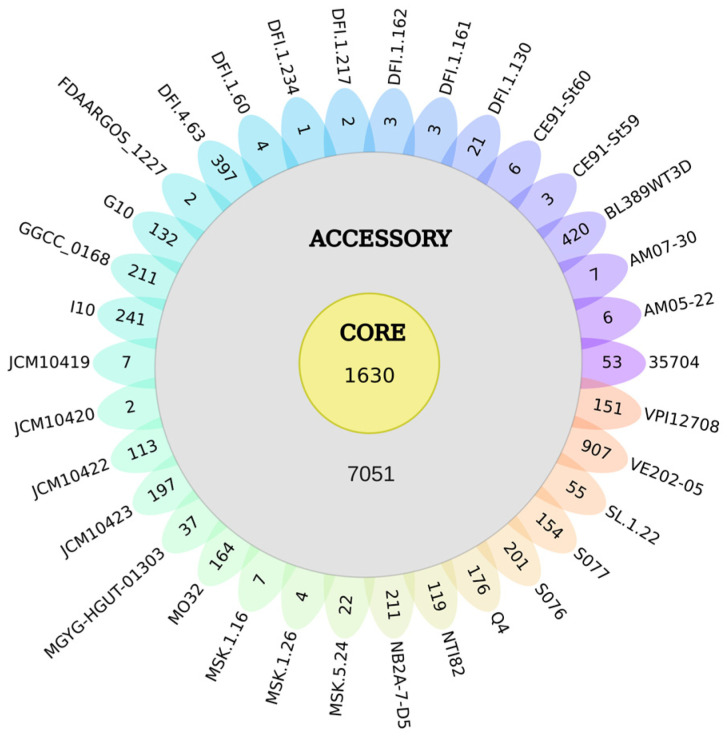
Genetic diversity of *C. scindens*. The flower plot shows the size of the core genome, accessory genome, and unique genes of the 34 *C. scindens* strains. The number of core gene clusters is represented by the yellow circle in the center of the flower (1630), the total number of accessory genes is represented by the gray circle (7051), and the genes unique to each strain are shown on each petal of the flower.

The Roary analysis identified a pangenome containing 12,720 gene families, distributed in the core genome, accessory genome, and unique or strain-specific genes. We define a ‘core’ gene as a gene found in all genomes analyzed. A total of 1630 gene groups are in the core, representing almost 13% of the total pangenome, with 7051 accessory groups. Furthermore, 4039 strain-specific genes distributed among *C. scindens* strains are represented (Figure 2), where 19 strains have between 1 and 55 strain-specific genes, and 15 have more than 100 genes. The human-associated strain VE202-05 [65] has the highest number of unique genes with a total of 907 genes, followed by BL389WT3D with 420 genes, which might be due to its isolation from pig feces [66].

**Figure 3:**
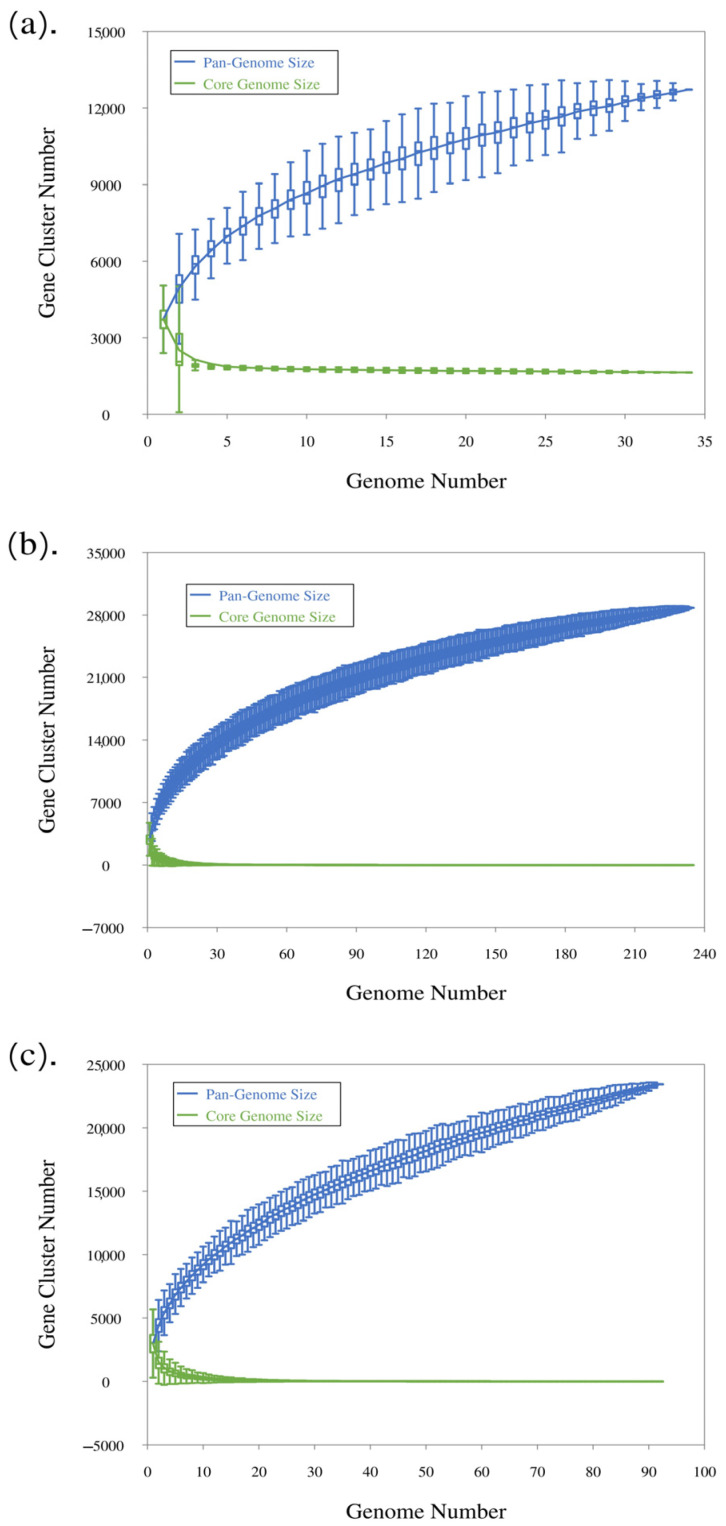
Pan−core plots for *C. scindens.* (**a**) Pan-core plot of the 34 cultured strains of *C. scindens.* (**b**) Pan-core plot including 200 *C. scindens* MAGs. (**c**) Pan-core plot including 58 *C. scindens* dereplicated MAGs. The graphics show cumulative curves of the upward trend in the number of pangenome gene families (in blue) and the downward trend of core gene families (in green) with each consecutive addition of a *C. scindens* genome. The rising curve in blue shows an open pangenome.

Analysis using a binomial mixture model as implemented in the function binomixEstimate from the micropan package yielded an estimate of core genome size of between 1356 (higher complexity model K = 6) and 1630 (lower complexity model, K = 3), which is very close to the number the curve of the core element seems to have stabilized at with 34 genomes (Figure 3a). This indicates that the essential set of genes for *C. scindens* has been identified and should not change significantly with the sequencing of more strains. The identification of the core genome allows us to infer characteristics such as the bacteria’s lifestyle, as this group of genes can encode resistance to antibiotics and heavy metals, cell wall components, virulence, and metabolic genes, among other information. On the other hand, strain-unique genes, which do not bear similarity to closely related strains, confer biological individuality, host specificity, and pathogenesis [30].

### 3.3. Pangenome Analysis After Addition of C. *scindens* Metagenome-Assembled Genomes

Pangenome analysis of *C. scindens* MAGs was determined considering the percentage of completeness of genomes, with analysis being carried out for each level of completeness. A group of 200 MAGs and another with 58 dereplicated MAGs were analyzed. To calculate the pangenome, the 34 previously described complete genomes of *C. scindens* were also included. The analysis shows that as MAG completeness decreases, the pangenome size tends to increase, while the number of gene groups in the core genome tends to be absent (Table 3 and Table 4); thus, sampling effects due to genome incompleteness severely affect the estimation of core size. After testing multiple completeness values with the 200 MAGs, the core genome was determined using MAGs with a completeness value of at least 85%. A large majority of the MAGs included in this study have completeness of at least 85% (Supplementary Figure S1). A total of 157 genomes are represented by a pangenome size of 19,189 gene families and a core genome of 132 gene groups, or almost 7% of the total pangenome (Table 3). Pangenomic analysis of MAGs can be affected by fragmentation, incompleteness, and contamination [67]. For example, it has been found that fragmentation and incompleteness lead to a significant loss of core genes, which translates into incorrect pangenomic functional predictions and inaccurate phylogenetic trees, and on the other hand, contamination influences the accessory genome. Accordingly, in our analyses, the core genome size was reduced by at least 50% for each increase of 5% in incompleteness (Table 3). The use of a higher completeness threshold, such as 95 or 90%, is recommended when defining core genes and carrying out a pangenome analysis combining complete genomes and MAGs. In the current study, we avoided this problem by using only the 34 genomes from cultivated strains in order to define the core genome, since we have a good sampling of such well-assembled and complete genomes. The MAGs were used mostly to investigate the accessory genome, which kept growing with the addition of new MAGs (Figure 3b,c).

### 3.4. Pangenome Profile

The plots provided by Roary and PanGP show the relationship between the pangenome, core genome, and the number of genomes, along with the different gene family distribution within the genomes under study, allowing us to estimate the pangenome profile as open or closed. Overall, the number of gene families in the pangenome and core genome increases and decreases, respectively, with each consecutive addition of a *C. scindens* genome, as expected.

According to the analysis of the 34 cultured *C. scindens* strains, the pangenome profile is open (Figure 3a), since as the number of sequenced genomes increases, the total number of gene families also increases. This is confirmed by the application of the Heaps’ Law formula, which results in an alpha value of 0.845, consistent with an open pangenome [68]. The core genome, on the other hand, seems to have stabilized. Likewise, after adding 200 total MAGs and dereplicated MAGs of *C. scindens*, the pangenome profile remains open, with a Heaps’ alpha of 0.768, and the core genome becomes nearly absent (Figure 3b,c), due to genome incompleteness, as discussed above.

### 3.5. Identification of C. scindens Strain Groups

**Figure 4:**
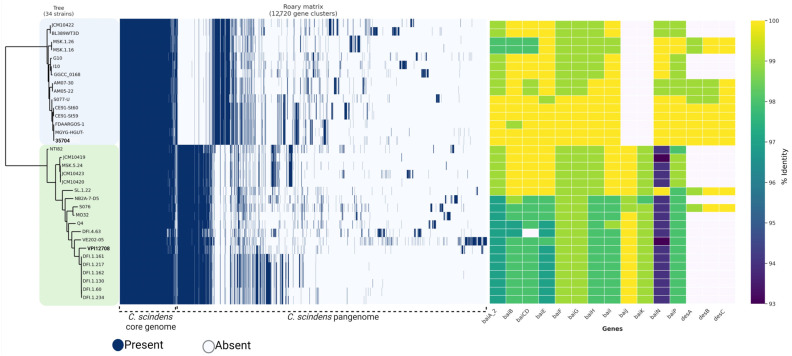
Pangenomic comparison of the 34 *C. scindens* strains. On the left: Phylogenomic tree and matrix of presence (blue lines) and absence (white lines) of core and accessory genes of the pangenome. The phylogenomic tree was generated based on 1490 single-copy orthologous genes shared by the 34 genomes of *C. scindens.* The tree was inferred using the maximum likelihood method by running 1000 fast bootstrap pseudoreplicates. The clades formed were referred to as group 1 and group 2, marked on the tree by light blue and green boxes, respectively. The main representative strains for the two groups are marked in bold typeface. On the right side: Heatmap of *bai* and *des* genes in the 34 *C. scindens* genomes. The percentage value of sequence identity is shown in color, with the highest value in yellow and the lowest in purple. The lack of the gene is indicated by a white rectangle.

We utilized Roary to group the 12,720 gene families of the pangenome into a presence/absence matrix and a phylogenomic tree based on the core genes, identifying a relationship between the tree and the distribution of core and accessory gene families among the 34 *C. scindens* strains (Figure 4). These strains were separated into two phylogenomic groups, referred to here as “group 1” (the *C. scindens* ATCC 35704 group) and “group 2” (the *C. scindens* VPI 12708 group).

**Figure 5:**
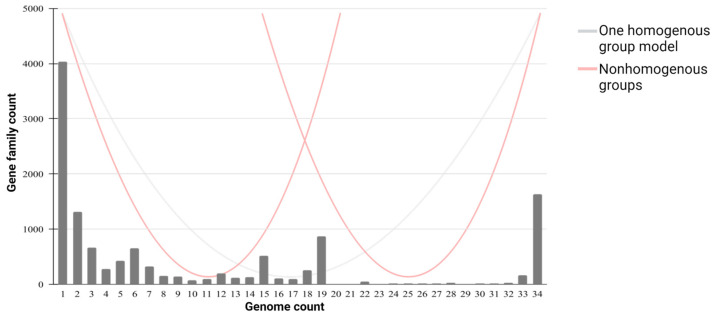
Gene frequency versus number of *C. scindens* genomes. The graph shows the frequency of gene clusters in the 34 *C. scindens* genomes. The left bar “1” represents the number of strain-specific genes, the right bar “34” indicates the core genome, and the central bars refer to the accessory genome. Lines are drawn to show the theoretical distribution of a “U” shaped “homogeneous” distribution (gray) and a “W” shaped distribution (red) for a “non-homogeneous” plot.

To represent a pangenome appropriately, the gene frequency spectrum function G(k) needs to be considered, which is defined as the number of orthologous groups containing genes from exactly k genomes [69]. Generally, in a set of strains belonging to the same species, the spectral function of the pangenome is U-shaped, without internal peaks that differentiate the number of gene groups found in the genomes. However, in a mixed sample, the spectrum function will have internal peaks. In other words, it is defined as “homogeneous” if the set of genomes presents a U-shaped spectrum function, and as “non-homogeneous” if the set contains internal peaks [69]. In our analysis of 34 cultured strain genomes, the number of genes specific to one strain is shown on the left (bar “1”), the core genome shared by all 34 strains is shown on the right, and the accessory genome is shown in the bars in between, present in 2 to 33 genomes (Figure 5). Our plot does not present the U-shape associated with a homogeneous distribution that would arise from the analysis of a single species but rather presents internal peaks that split the graph into two main regions. This, associated with the gene content and phylogenomic analyses, suggests the presence of two different groups of *C. scindens* strains.

### 3.6. Average Nucleotide Identity Analysis of 34 C. *scindens* Strain Cultivars

The average percentage nucleotide identity calculated by the PyANI tool using the BLAST ANIb method provides the genomic relationship and variation level between the recently sequenced *C. scindens* genomes and the genomes obtained from NCBI, represented by color intensities based on the calculated percentage of identity (Supplementary Figure S2). The 95% identity threshold value for species separation in an ANI analysis was used here, as suggested in the literature [56]. Our results show two groups divided into 15 and 19 strains with a difference of approximately 4–5% in their genomic sequences. Identity within each group is ≥98%, while identity between groups is 94.5 to 96%, whereas with the *C. hylemonae* genome, the identity values are between 74 and 76%. The present ANI values of *C. scindens* suggest the presence of two possible distinct microbial species or at least an ongoing speciation process.

These results provide a basis for understanding the formation of the two groups of *C. scindens* strains, group 1 and group 2 (Figure 4), as previously demonstrated in the pangenome analysis, thus allowing further elucidation of the relationship between the genomes.

### 3.7. SSU rDNA Analysis of 34 C. *scindens* Strain Cultivars

We have analyzed the SSU rRNA genes from the 34 strains of *C. scindens* and outgroup *C. hylemonae* TN271, identifying all ribosomal cistrons, running their maximum likelihood phylogeny, and calculating pairwise sequence distances. With the exception of two strains, all complete or nearly complete genome sequences (i.e., those containing five contigs or fewer, as shown in Table 1) presented four copies of the complete ribosomal cistron. The two exceptions, strains CE91-St59 and CE91-St60, presented three copies each of the set of ribosomal genes. Except for strain MGYG-HGUT-01303, which had two copies, all other incomplete genomes presented just one copy of the ribosomal cistron. In addition, strains SL.1.22, MSK.1.16, and VE202-05 lacked a portion of the beginning of the SSU rRNA gene sequence, strain MSK.1.26 lacked part of the end, and strain DFI.1.161 had sequence missing both from the start and the end of the gene (Supplementary File S1).

The distance values between all *C. scindens* SSU rRNA sequences (Supplementary Figure S3 and File S2), expressed in terms of the uncorrected (i.e., directly observed) number of differences per 100 nucleotides, revealed an interesting pattern, with two different kinds of SSU rRNA gene sequences present in some strains, but not all of them. All SSU rRNA copies within *C. scindens* strains in group 1 (the ATCC 35704 group) genomes belonged to the same sequence type, i.e., they were all very similar, with at most 0.20% difference (average: 0.05%) in pairwise comparisons; the SSU sequences of different strains were often identical, in fact.

Among strains from group 2 (the VPI 12708 group), the picture is more complex when we look at the SSU rRNA sequence differences, presenting two distinct ranges of dissimilarity present: 0.00 to 0.46% (type A SSU rRNA, average: 0.17%) and 0.85 to 1.18% (type B, average: 1.00%) (Supplementary File S2). Almost all of the differences between the two different kinds of SSU rRNA genes found in group 2 are situated between positions 75 and 115 of the alignment (Supplementary File S1). As can be seen in the alignment, type B has more similarities with the group 1 SSU rRNA sequences in that region of the molecule than with type A that is also present in some genomes of group 2 strains. Strains VPI 12708 and MO32 presented two copies of each SSU type, and strain S076 presented three copies of type A and three copies of type B. Of the group 2 strains with more fragmented genome assemblies, all of which had only one ribosomal gene set identified, seven of them (VPI 12708, SL.1.22, DFI.1.130, DFI.1.161, DFI.1.127, DFI.1.234, and DFI.1.60) presented type B sequences, while two (DFI.1.162 and DFI.4.63) presented type A sequences. Due to the incompleteness of these genome sequences, it cannot be ruled out that some (or even all) of them possess both SSU sequence types.

The distances between group 1 and group 2 sequences agree with the presence of two types of SSU rRNA gene in group 2 genomes, with two ranges of distances present: 0.00 to 0.42% (average: 0.27%) and 1.04 to 1.39% (average: 1.19%). The traditionally suggested threshold value to separate bacterial species using the complete SSU rRNA gene is ≥3%. Therefore, this gene does not reflect the possible ongoing speciation process as closely as the ANI results described above, since SSU rRNA gene distances never went above 1.39%.

The relatively short distances between group 2’s type B SSU sequences and those found in group 1 are reflected in the phylogenetic tree of the SSU rRNA genes when all copies are included (Supplementary Figure S4). By rooting the tree on the branch leading to *C. hylemonae*’s sequence, it can be seen that all group 1 sequences seem to have originated from an internal branch of group 2’s type B SSU rRNA, with its most closely related sequence being one of the copies from strain MO32. The branch lengths on the tree also show that there is much more sequence variation within the sequences from group 2 than those of group 1, indicating that group 1 might have gone through a relatively recent population bottleneck. Both observations suggest that group 1 probably descends from a subset of group 2 that specialized in a different niche in the host. However, given the overall very low diversity in the SSU rRNA sequences (Supplementary File S1), leading to low phylogenetic signal (and therefore low bootstrap support values), these hypotheses are currently tentative. Indeed, the highly supported phylogenomic analysis does not place the strains in the same manner. Further investigation, including more strains and focusing on more variable genes, will be needed in order to better understand the origin of the two different *C. scindens* groups.

### 3.8. COG Distributions of C. *scindens* Core, Accessory, and Unique Genes

**Figure 6:**
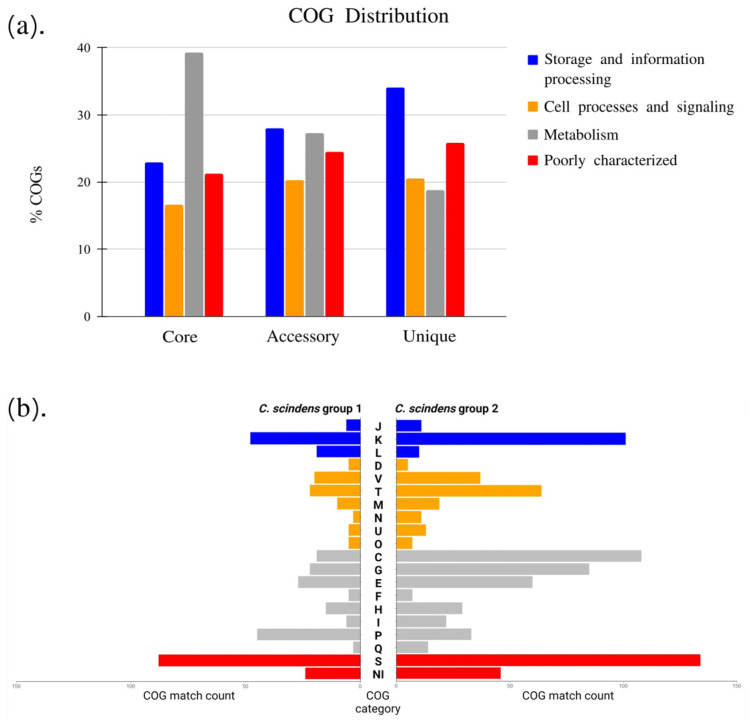
Distribution of core, accessory, and unique genes in clusters of orthologous groups (COG). (**a**) The distribution of the main functional categories of the pangenome. (**b**) COG analysis of the accessory genome of Group 1 and Group 2 (see Figure 4 phylogenomic analysis).

The distribution of functional genes from the core, accessory, and unique gene families into COG categories shows that the main ones are constituted by genes for storage and information processing (classes J, K, and L), cellular processes and signaling (classes D, M, N, O, T, U, and V), metabolism (classes C, E, F, G, H, I, P, and Q), and poorly characterized genes (class S) (Figure 6a). Determining the functional classification of the core genome through COG categories is important, as these genes are responsible for the most fundamental biological characteristics. The analysis of core genes shows 39% of genes related to metabolism, a higher percentage compared to the other categories. On the other hand, unique genes show a greater number for the storage and information processing category (34%), followed by unknown or poorly characterized gene function (26%).

Among the genes associated with metabolism, the COG annotation of the core genes shows that category C (energy production and conversion) is the most common metabolic function (8.5%), followed by categories E (6.6%) and H (6.2%). Category K, belonging to the category of information storage and processing, presented 9.7% of genes in the core genome. The category with the highest number of gene families in the pangenome, as expected from previous studies, is category S (unknown function). The COG annotation of accessory genes and unique genes shows greater numbers in categories K and L (27% and 32%, respectively). Similar results from functional annotation of gene families have been reported in pangenome analyses in other pathogenic and non-pathogenic *Clostridium* species, such as *C. perfringens* [70], *C. butyricum* [71], and *C. botulinum* [30].

### 3.9. KEGG Pathway Distributions of the C. *scindens* Pangenome

KAAS functional annotation results for the comparative analysis of protein sequences representative of the core, accessory, and unique genome of *C. scindens* against the KEGG database show a greater gene distribution percentage for metabolism-related pathways (Supplementary Figure S5A). These data coincide with our previous result of COG distributions related to metabolic function. Environmental information processing genes are the second-highest category, followed by the genetic information processing category.

In the core genome, carbohydrate metabolism and amino acid metabolism are the two subcategories with the highest number of genes (Supplementary Figure S5B), where the pentose–phosphate pathway, and alanine and glycine metabolism pathway collect the highest number of core genes with 18 and 20 genes, respectively. In the accessory and unique genome, carbohydrate metabolism, amino acid metabolism, and membrane transport are the predominant subcategories. In the second-largest main category, processing of environmental information, there is a greater number of accessory and unique genes in the membrane transport subcategory, where the ABC transporter pathway owns the largest number of genes, presenting 73 accessory genes and 42 unique genes.

Furthermore, the KEGG distribution was determined in categories and subcategories of non-core shared genes by the groups of strains identified above as group 1 and group 2. Overall, no full pathways were found to be exclusive to one of the groups, but most genes complement or enrich main core genome metabolic pathways. Group 2 has a higher proportion of genes for metabolism, while group 1 has a higher proportion of genes for the environmental information processing category (Supplementary Figure S6A). Additionally, group 1 presented the largest number of genes for the membrane transport subcategory, and group 2 for the carbohydrate metabolism subcategory (Supplementary Figure S6B). Quorum sensing proteins seem to be more prevalent in group 1, suggesting more efficiency in cell–cell communication and response to microbial competition.

### 3.10. Identification of Bile-Acid-Metabolizing Genes in C. *scindens* Genomes

**Figure 7:**
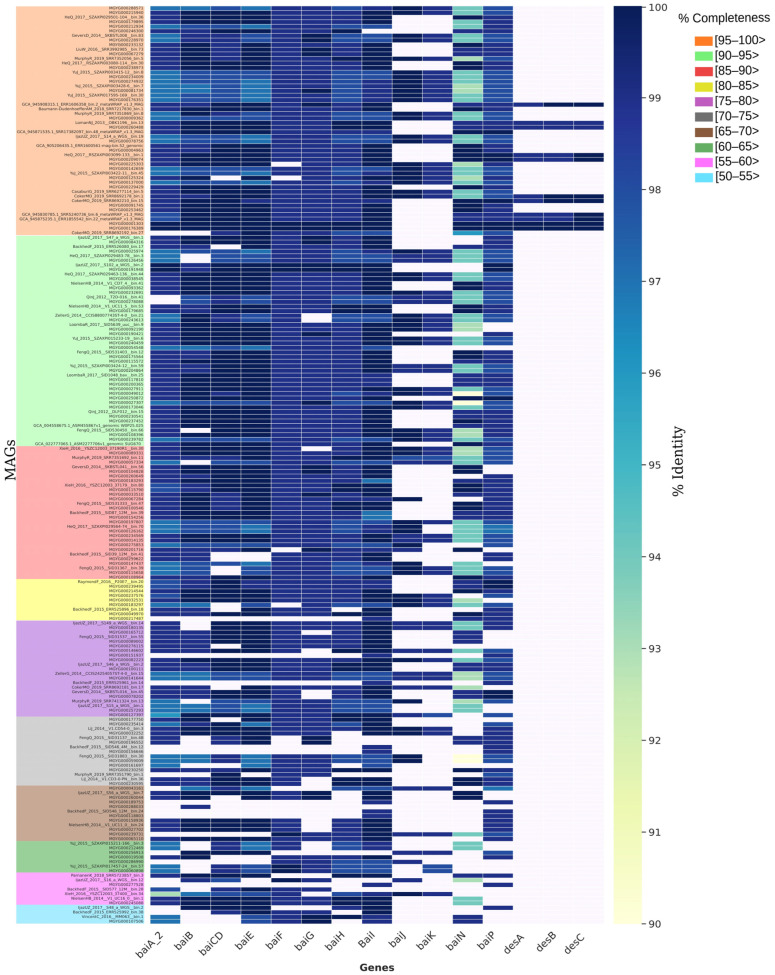
Heatmap of *bai* and *des* genes present in 200 *C. scindens* MAGs. The percentage value of sequence identity is shown in color, with the highest value in dark blue and the lowest in light yellow. The absence of the gene is indicated by the color white. The completeness of the genomes is represented in descending order and indicated by the colored boxes.

To determine the presence of *bai* and *des* genes, BLASTP alignments with an identity greater than 90% and an e-value of 0.0 of the 34 *C. scindens* strain cultivars and MAG proteomes were considered (Figure 4 and Figure 7). The results suggest that genes located in the *baiABCDEFGHI* operon of *C. scindens* are highly conserved in their amino acid sequences among different strains. The *baiJ* and *baiK* genes (part of the *bai* regulon) are not present in the 15 strains in group 1, and the *baiCD* gene was absent in the DFI.4.63 strain from group 2, since this gene is fragmented. This fragmentation could be due to sequencing or assembly errors, but only resequencing of the relevant genomic region in this strain will be able to tell. On the other hand, 10 strains from group 1 feature *desA, desB,* and *desC* genes, while only two strains from group 2 contain these same genes (Figure 4).

Previously, comparative analysis of the *bai* genes in *Eubacterium* sp. c-25 and other DCA-producing species including *C. scindens* strains ATCC 35704 and G10 [72] showed that the sequence identity of the predicted Bai proteins from *Eubacterium* is low (43–55%) compared to *C. scindens*, with the exception of BaiH (83%). This suggests that *baiH* has a key role in 7α-dehydroxylation and is a required protein. The 7α-dehydroxylation of primary bile acids is a multistep pathway that involves a group of *bai* genes that encode enzymes that participate in this process [73]. Furthermore, bile acids (BAs) are important due to their relationship with intestinal diseases, such as CRC. The distribution of *bai* genes among *Bacillota* was observed in human gut metagenomes, as determined through a phylogenetic analysis and searches in hidden Markov models, resulting in a greater abundance of *baiCD*, *baiE*, *baiJ*, and *baiP* genes in CRC patients compared to healthy subjects [74]. The diversity of the presence and absence of *bai* and *des* genes in MAGs shows absence of *baiJ* and *baiK* genes in most genomes, with *desA*, *desB*, and *desC* genes almost completely absent (Figure 7). The genes involved in the biotransformation of BAs in *C. scindens* are the genes of the *baiABCDEFGHI* operon in addition to *bai* regulon genes *baiJKLM* [74,75], and the *baiN* gene. The *baiN* gene encodes a protein from the flavoprotein family involved in the BA 7α-dehydroxylation pathway [76]; the sequence of this protein is conserved among 7α-dehydroxylating bacteria closely related to *C. scindens*. The initial characterization of *baiN* in *C. scindens* ATCC 35704 and the presence of the gene in *C. hylemonae* were reported by Harris and colleagues [76]. We then determined *bai* and *des* gene frequency in 200 MAGs of *C. scindens* (Figure 7). The *bai* regulon genes were found in all MAGs, with more gaps as the genome completeness was reduced to ≥90%. Interestingly, the *desABC* genes were identified in only 20 *C. scindens* MAGs.

Vital and collaborators showed the diversity of intestinal bacteria with *bai* genes, using BLAST to search for possible homologues of the *baiN* gene in a set of MAGs of the *Lachnospiraceae* family and in genomes of isolates available in databases, using as a reference the *bai* sequences from *C. scindens* ATCC 35704 [77]. In that study, *baiN* was identified in most genomes; however, amino acid identities were low even for *Clostridium hiranonis*, a verified 7α-dehydroxylating bacterium. Furthermore, the authors propose the absence of genetic sequences homologous to the *baiN* gene outside the main clade of *Lachnospiraceae* that includes *C. scindens* and *C. hylemonae*. By contrast, we located *baiN* in 168 MAGs and all 34 isolated strain genomes (Figure 4 and Figure 7).

The *baiJ* and *baiK* genes were discovered in a multigene operon conserved in *C. scindens* VPI 12708 and *C. hylemonae* [75]. The operon encodes a bile acid CoA transferase (*baiK*) and a predicted flavin-dependent oxidoreductase (*baiJ*); however, this pathway is not found in all 7α-dehydroxylating strains of *C. scindens*, as shown in our results (Figure 4). The *baiP* gene serves a similar function to *baiJ* in the formation of allo-secondary bile acids [74,78]. The *baiP* gene was identified in 186 *C. scindens* MAGs and all 34 isolated strain genomes. These data indicate that *C. scindens* strains are universal in their potential to generate allo-secondary bile acids.

### 3.11. Predicted Metabolic Pathways in the Core Genome

**Figure 8:**
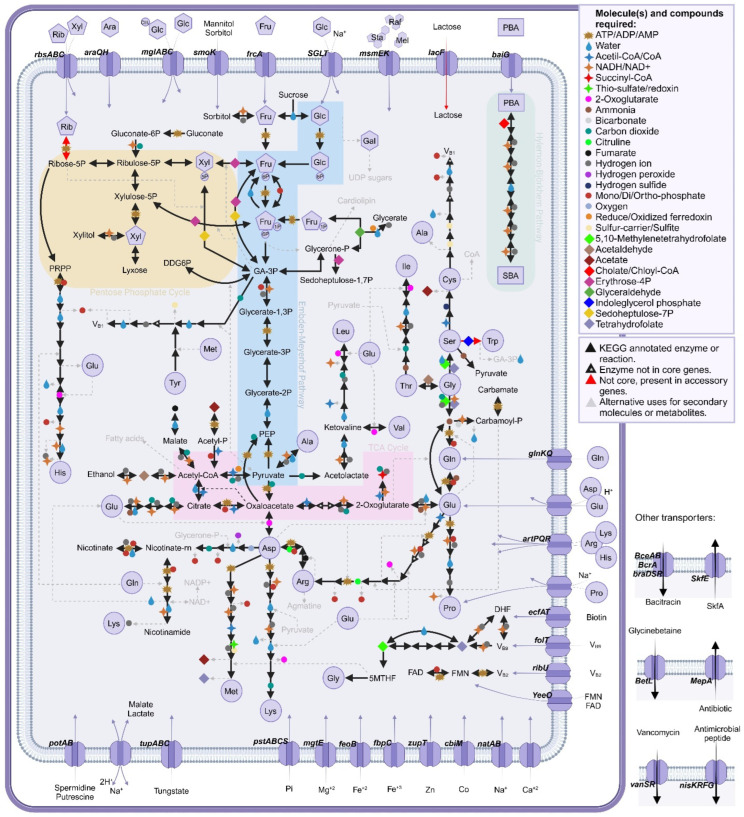
Key metabolic pathways and transporters represented in the core genome of *C. scindens.*

We previously developed a defined growth medium for *C. scindens* ATCC 35704 using the “leave-one-out” approach to identify essential vitamins and amino acids [11]. With this approach, we determined that the amino acid tryptophan and three vitamins (riboflavin, pantothenate, and pyridoxal) were required for the growth of *C. scindens* ATCC 35704. In this medium, *C. scindens* ATCC 35704 fermented glucose mainly to ethanol, acetate, formate, and H_2_. However, it is not clear whether other strains of *C. scindens* have similar nutritional requirements or fermentation end products. We therefore examined the core genome, particularly with respect to predicted amino acid and vitamin metabolism, determining the metabolic pathways located in the core genome of the 34 genomes of cultured *C. scindens* strains (Figure 8).

*C. scindens* has a complete glycolytic pathway, a complete pentose phosphate pathway, and a “horseshoe” TCA cycle from oxaloacetate to succinyl~SCoA. Oxaloacetate is generated from phosphoenolpyruvate, and malate and fumarate from pyruvate. The complete HB pathway is a core feature of *C. scindens* strains, but steroid-17,20-desmolase, while present in genomes from both groups, is not part of the core genome. The core genome contains complete pathways for the biosynthesis of the majority of amino acids. L-histidine and L-alanine are predicted to be obtained from carnosine metabolism. As part of the accessory genomes of both groups 1 and 2, a tryptophan synthase enzyme (EC 4.2.1.20) was annotated, suggesting the possibility that L-tryptophan might be synthesized from indole and L-serine, which is a major product of L-tryptophan catabolism by gut microbiota [79]. The core and accessory genomes contain the near complete shikimate pathway for the biosynthesis of L-phenylalanine and L-tyrosine.

Pantothenate biosynthesis is not part of the core; however, a pathway from pantothenate to CoA was evident in the core genome (Figure 8). Synthesis of R-pantothenic acid requires B-alanine, a molecule that seems to be generated by group 1 from L-aspartate, but not by group 2. Pathways from nicotinate to NAD+ and NADP+ from L-aspartate are present. Thiamine and cobalamin biosynthesis pathways are part of the core genome. Genes encoding enzymes involved in one carbon pool by folate are complete. Genes encoding enzymes involved in converting riboflavin to FMN and FAD are present; however, biosynthesis pathways for riboflavin are absent. Folate is required, although the one carbon pool by folate cycle is present in the core genome. The complete biosynthetic pathway for thiamine is present. Lipoate salvage, but not biosynthesis, is present in the core genome. Thus, supplementation of our previous defined medium for *C. scindens* ATCC 35704 [11] with a complete set of vitamins (with exception of thiamine) and additional amino acids (tryptophan, phenylalanine, and tyrosine) is advisable.

The current study represents the first pangenome analysis of the important bile acid and steroid-metabolizing gut microbial species *Clostridium scindens.* In a phylogenetic analysis of the rRNA SSU sequences and the core genome of members of the *Lachnospiraceae* family, including species identified as *Clostridium* related to the genus *Lachnoclostridium*, it was demonstrated that *C. scindens* was not grouped with other *Clostridium* isolates, despite being classified as a member of this genus; these results showed that all members of the genus *Lachnoclostridium* analyzed formed a distinct monophyletic group, with the exception of *C. scindens*, which is more closely related to isolates of the genus *Dorea*, which includes intestinal commensal species [80] and has members that also harbor the *bai* operon [72,81,82].

We have shown by whole-genome phylogeny, gene content, and genome-wide similarity analysis that strains of *C. scindens* actually group into two distinct clades, group 1 (ATCC 35704) and group 2 (VPI 12708), thus supporting Bokkenheuser’s claims of “taxonomic value” for steroid-metabolizing activities and that *C. scindens* VPI 12708 and *C. scindens* ATCC 35704 would be considered distinct and separate species. *C. scindens* VPI 12708, isolated in the 1970s, and *C. scindens* ATCC 35704, isolated in 1985, are both known for bile acid dehydroxylation, but unlike *C. scindens* VPI 12708, *C. scindens* ATCC 35704 is also capable of side-chain cleavage, via steroid-17,20-desmolase, of 17α,21-dihydroxysteroids (e.g., cortisol, tetrahydrocortisol, cortisone, 11-deoxycortisol, and 21-deoxycortisol). Bokkenheuser and associates who isolated *C. scindens* ATCC 35704 argued that steroid-metabolizing activities (e.g, 17, 20-desmolase activity) are species-specific and as such represent distinctive traits that are useful in bacterial identification and taxonomy. However, in 2000, ”*Eubacterium* VPI 12708” and five additional bile acid dehydroxylating strains were all reclassified as *C. scindens* based on carbohydrate fermentation profiles, 16S rRNA sequencing, and DNA–DNA similarity tests. Steroid-metabolizing activities, other than bile acid dehydroxylation (i.e., presence or absence of *bai* genes), were not considered in their strain reassignment. What the taxonomic designation should be for *C. scindens* VPI 12708 and related strains (group 2) awaits determination.

Understanding the functional consequences of strain variation in host-associated microbiomes is a current focus in the field, particularly in building microbial consortia for therapeutic applications [83,84,85]. Bile acid metabolite profiles are important in a myriad of considerations in the germination and vegetative growth of *C. difficile* [86], immune cell differentiation and function in inflammatory bowel disease [7,87], neurodegenerative conditions [88], and metabolic disorders [89,90]. Thus, understanding the depth and breadth of strain variations among potential Live Biotherapeutic Product candidate species is important in advancing microbiome engineering.

## 4. Conclusions

Our results reveal that the *C. scindens* pangenome is open, containing, for the set of 34 cultured strains, 12,720 groups of gene families, distributed in 1630 groups of core genes, 7051 accessory groups, and 4039 unique strain groups, thus indicating the existence of genomic diversity that could be discovered with additional strain isolation and sequencing. Furthermore, the inclusion of 200 *C. scindens* MAGs did not close the pangenome, revealing the high plasticity in the accessory genomes of this species. The inference, by phylogenetic, gene content, and distance methods, of two separate groups of isolates was seen among the 34 cultured strains, which differ by approximately 4–5% in their genomic sequences and in the presence of some genes from the *bai* operon. These two clades might represent species that have diverged very recently or, at the very least, an ongoing speciation process caught on the verge of completion. Functional annotation of the pangenome determined that metabolism is the predominant functional category. In summary, the analysis of the structure and function of the *C. scindens* pangenome provides new data on the genomic content and variability between strains of this species, which will hopefully help to stimulate future research on this important intestinal bile-acid-metabolizing bacterium.

## Figures and Tables

**Table 1:** Characteristics and genomic information for 34 cultured strains of *C. scindens*.

Strain	Contigs or Scaffolds (Genome Assembly Level)	Accession/RefSeq Number	Host (Source)	Geographic Origin	BioSample	BioProject	Genome Assembly Identifier
JCM10419	1 (not circularized)	CP137824	*Homo sapiens* (feces)	Japan	SAMN37482747	PRJNA1026650	ASM3353943v1
JCM10420	1 (closed)	CP137823	*Homo sapiens* (feces)	Japan	SAMN37482748	PRJNA1026650	ASM3353941v1
JCM10422	2 contigs	CP137821-CP137822	*Homo sapiens* (feces)	Japan	SAMN37482749	PRJNA1026650	ASM4093202v1
JCM10423	1 (not circularized)	CP137820	*Homo sapiens* (feces)	Japan	SAMN37482750	PRJNA1026650	ASM3353951v1
I10	1 (closed)	CP137819	*Homo sapiens* (feces)	Japan	SAMN37482751	PRJNA1026650	ASM3353949v1
MO32	1 (closed)	CP137818	*Homo sapiens* (feces)	Japan	SAMN37482752	PRJNA1026650	ASM3353945v1
NT182	1 (closed)	CP137817	*Homo sapiens* (feces)	Japan	SAMN37482753	PRJNA1026650	ASM3353953v1
S076	1 (closed)	CP137816	*Homo sapiens* (feces)	Japan	SAMN37482754	PRJNA1026650	ASM3353947v1
S077	5 contigs	CP137811-CP137815	*Homo sapiens* (feces)	Japan	SAMN37482755	PRJNA1026650	ASM4093201v1
VPI12708	1 (closed)	CP113781	*Homo sapiens* (feces)	Germany	SAMN31775693	PRJNA902789	ASM2794165v1
CE91—St59	1 (closed)	AP025569.1	*Homo sapiens* (feces)	Japan	SAMD00389867	PRJDB11902	ASM2284581v1
CE91—St60	1 (closed)	AP025570.1	*Homo sapiens* (feces)	Japan	SAMD00389868	PRJDB11902	ASM2284583v1
G10	1 (closed)	AP024846.1	*Rattus norvegicus* (cecal content)	Japan	SAMD00239677	PRJDB10323	ASM2089211v1
Q4	1 (closed)	CP080442.1	*Homo sapiens* (feces)	USA	SAMN20488193	PRJNA750754	ASM1959792v1
BL389WT3D	1 (closed)	CP045695.1	*Sus scrofa domesticus* (feces)	Germany	SAMN13152203	PRJNA561470	ASM968469v1
FDAARGOS_1227	1 (closed)	CP069444.1	Not available	USA	SAMN16357369	PRJNA231221	ASM1688900v1
ATCC 35704	1 (closed)	CP036170.1	*Homo sapiens* (feces)	USA	SAMN10519000	PRJNA508260	ASM429512v1
AM05-22	56 scaffolds	GCF_027662895.1	*Homo sapiens* (feces)	China	SAMN31808509	PRJNA903559	ASM2766289v1
AM07-30	50 scaffolds	GCF_027662765.1	*Homo sapiens* (feces)	China	SAMN31808516	PRJNA903559	ASM2766276v1
SL.1.22	52 contigs	GCF_020555615.1	*Homo sapiens* (feces)	USA	SAMN22167568	PRJNA737800	ASM2055561v1
DFI.1.234	107 contigs	GCF_022137935.1	*Homo sapiens* (feces)	USA	SAMN24725968	PRJNA792599	Not available
GGCC_0168	256 contigs	GCF_017565985.1	*Homo sapiens* (feces)	USA	SAMN14737934	PRJNA628657	ASM1756598v1
DFI.1.217	96 contigs	GCF_020562885.1	*Homo sapiens* (feces)	USA	SAMN22167352	PRJNA737800	ASM2056288v1
DFI.1.162	125 contigs	GCF_020563365.1	*Homo sapiens* (feces)	USA	SAMN22167324	PRJNA737800	ASM2056336v1
DFI.1.161	160 contigs	GCF_024463895.1	*Homo sapiens* (feces)	USA	SAMN28944463	PRJNA792599	ASM2446389v1
MSK.1.26	93 contigs	GCF_013304105.1	*Homo sapiens* (feces)	USA	SAMN14067588	PRJNA596270	ASM1330410v1
DFI.1.60	197 contigs	GCF_020561885.1	*Homo sapiens* (feces)	USA	SAMN22167389	PRJNA737800	ASM2056188v1
MSK.1.16	93 contigs	GCF_013304115.1	*Homo sapiens* (feces)	USA	SAMN14067587	PRJNA596270	ASM2056188v1
MSK.5.24	21 contigs	GCF_013304085.1	*Homo sapiens* (feces)	USA	SAMN14067589	PRJNA596270	ASM1330408v1
DFI.1.130	797 contigs	GCF_020563525.1	*Homo sapiens* (feces)	USA	SAMN22167316	PRJNA737800	ASM2056352v1
DFI.4.63	195 contigs	GCF_020560435.1	*Homo sapiens* (feces)	USA	SAMN22167449	PRJNA737800	ASM2056043v1
MGYG-HGUT-01303	41 scaffolds	GCF_902373645.1	*Homo sapiens* (feces)	Not available	SAMEA5850806	PRJEB33885	MGYG-HGUT-01303
NB2A-7-D5	39 contigs	GCF_024125195.1	*Homo sapiens* (feces)	Not available	SAMN28102059	PRJNA835435	ASM2412519v1
VE202-05	102 contigs	Not available	*Homo sapiens* (feces)	Japan	SAMD00004073	PRJDB524	ASM47184v1

**Table 2:** Genomic characteristics of 34 *C. scindens* strains.

	Strain	G + C%	tmRNA	tRNA	rRNA	CDS	Genome Size (bp)	No. Contigs
1	I10	46.5	2	56	12	3602	3,435,295	1
2	JCM10419	47.5	1	57	12	3682	3,940,699	1
3	JCM10420	47.4	1	57	11	3768	4,020,045	1
4	JCM10422	46.6	1	56	12	3390	3,501,106	2
5	JCM10423	46.9	1	57	11	4057	4,306,053	1
6	MO32	47.8	1	57	11	3691	3,929,075	1
7	NTI82	47.1	1	59	11	4137	4,318,168	1
8	S076	47.3	1	58	11	4103	4,290,604	1
9	S077	46.5	2	57	12	3386	3,403,497	5
10	VPI 12708 *	47.7	1	56	12	3716	3,983,052	1
11	ATCC 35704 *	46.3	2	58	12	3656	3,658,040	1
12	BL389WT3D *	45.5	2	58	12	3655	3,785,527	1
13	CE91-St59 *	45.9	2	56	12	3602	3,608,085	1
14	CE91-St60 *	45.9	2	56	12	3608	3,608,087	1
15	FDAARGOS_1227 *	46.3	2	57	12	3603	3,619,096	1
16	G10 *	46.6	2	57	12	3295	3,315,593	1
17	Q4 *	47.7	1	57	12	3725	3,941,835	1
18	AM05-22 *	46.5	2	54	3	3269	3,330,149	56
19	AM07-30 *	46.5	2	49	2	3272	3,331,670	50
20	DFI.1.130 *	46.9	1	53	4	4177	4,565,863	797
21	DFI.1.161 *	47.1	1	51	4	4041	4,325,545	160
22	DFI.1.162 *	46.9	1	52	4	4161	4,396,306	125
23	DFI.1.217 *	46.9	1	52	4	4161	4,389,871	96
24	DFI.1.234 *	47	1	52	4	4072	4,316,625	107
25	DFI.1.60 *	47.1	1	52	4	4047	4,309,437	197
26	DFI.4.63 *	47.5	1	52	4	3958	4,167,375	195
27	GGCC_0168 *	46.6	2	57	12	3390	3,417,088	256
28	MGYG-HGUT-01303 *	46.4	2	64	22	3574	3,622,605	41
29	MSK.1.16 *	46.4	2	54	4	3143	3,230,100	93
30	MSK.1.26 *	46.4	2	54	3	3140	3,227,969	93
31	MSK.5.24 *	47.5	1	54	4	3845	4,072,709	21
32	NB2A-7-D5 *	47.5	1	60	4	3980	4,182,602	39
33	SL.1.22 *	47.5	1	55	3	3706	3,970,092	52
34	VE202-05 *	47.8	0	48	2	4523	3,912,387	102

* Genomes previously sequenced and available in GenBank.

**Table 3:** Analysis results of the 200 *C. scindens* MAG pangenomes, depending on the completeness of the genomes used in the analysis. The size of the pangenome and *core* genome for each MAG completeness value is shown.

Completeness of MAGs %	Pangenome	*Core* Genome
≥95	14,625	850
≥90	17,713	401
≥85	19,189	132
≥80	19,792	75
≥75	21,923	8
≥70	22,864	4

**Table 4:** Pangenome analysis results of the dereplicated *C. scindens* MAG genomes.

Completeness of MAGs %	Pangenome	*Core* Genome
≥95	14,464	1120
≥90	15,144	931
≥85	16,168	501
≥80	16,629	276
≥75	19,083	25
≥70	20,023	6

## Data Availability

All scripts and HMMs used in the metagenomic analyses are available at https://github.com/AnantharamanLab/Clostridium_scindens_mining (accessed on 29 January 2025).

## Supplementary Materials

The following supporting information can be downloaded at: https://www.mdpi.com/article/10.3390/microorganisms13040857/s1, Figure S1: Completeness assessment of 200 MAGs; Figure S2: ANI analysis between the *C. scindens* genomes and the *C. hylemonae* genome; Figure S3: Distance matrix based on the SSU rRNA gene copies of the 34 *C. scindens* strains; Figure S4: Maximum likelihood phylogenetic tree of all SSU rRNA genes identified from 34 *C. scindens* genomes, with *C. hylemonae* as the outgroup; Figure S5: KEGG distribution of core, accessory and unique genes of the *C. scindens* pangenome; Figure S6: KEGG distribution of the groups of *C. scindens* strains, Group 1 and Group 2; Table S1: Accession numbers for all reference genes used in the Bai and Des protein searches; Table S2: CheckM results for 34 cultivated *C. scindens* strains, including the genomes recently published by our group and other complete and incomplete genomes from NCBI; Table S3: Genomic characteristics of 200 non-dereplicated *C. scindens* MAGs; Table S4: Genomic characteristics of 58 dereplicated MAGs; File S1: Sequence alignment generated by Muscle for all SSU rRNA genes identified in 34 *C. scindens* genomes and in *C. hylemonae*; File S2: Uncorrected distance matrix between all SSU rRNA gene sequences identified in 34 *C. scindens* genomes and in *C. hylemonae*, calculated by distmat of the EMBOSS suite.
