# Supplementary material for: Pangenome Analysis of Clostridium scindens: A Collection of Diverse Bile Acid- and Steroid-Metabolizing Commensal Gut Bacterial Strains
Source: Microorganisms. 2025 Apr 9;13(4):857. doi: 10.3390/microorganisms13040857 (PMC12029741; doi:10.3390/microorganisms13040857)
Supplement: Supplementary file 1 [file microorganisms-13-00857-s001.zip › microorganisms-3479729-supplementary.pdf]

Extended Data Figures and Tables

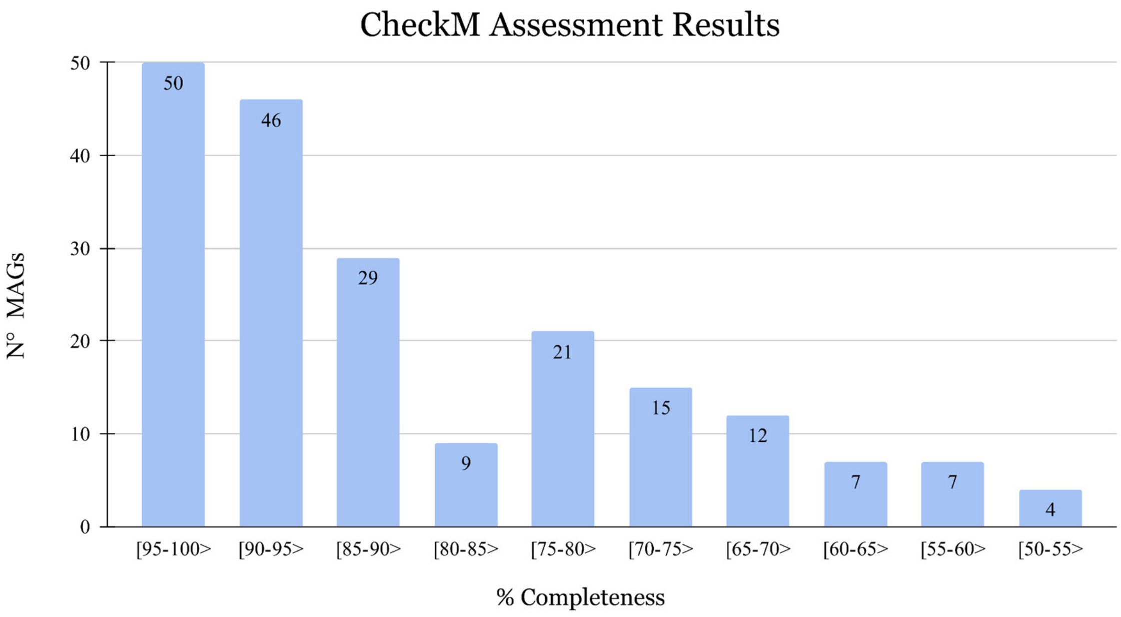

Supplementary Figure S1. Completeness assessment of 200 MAGs. Each bar represents a range of estimated completeness, in 5% increments.

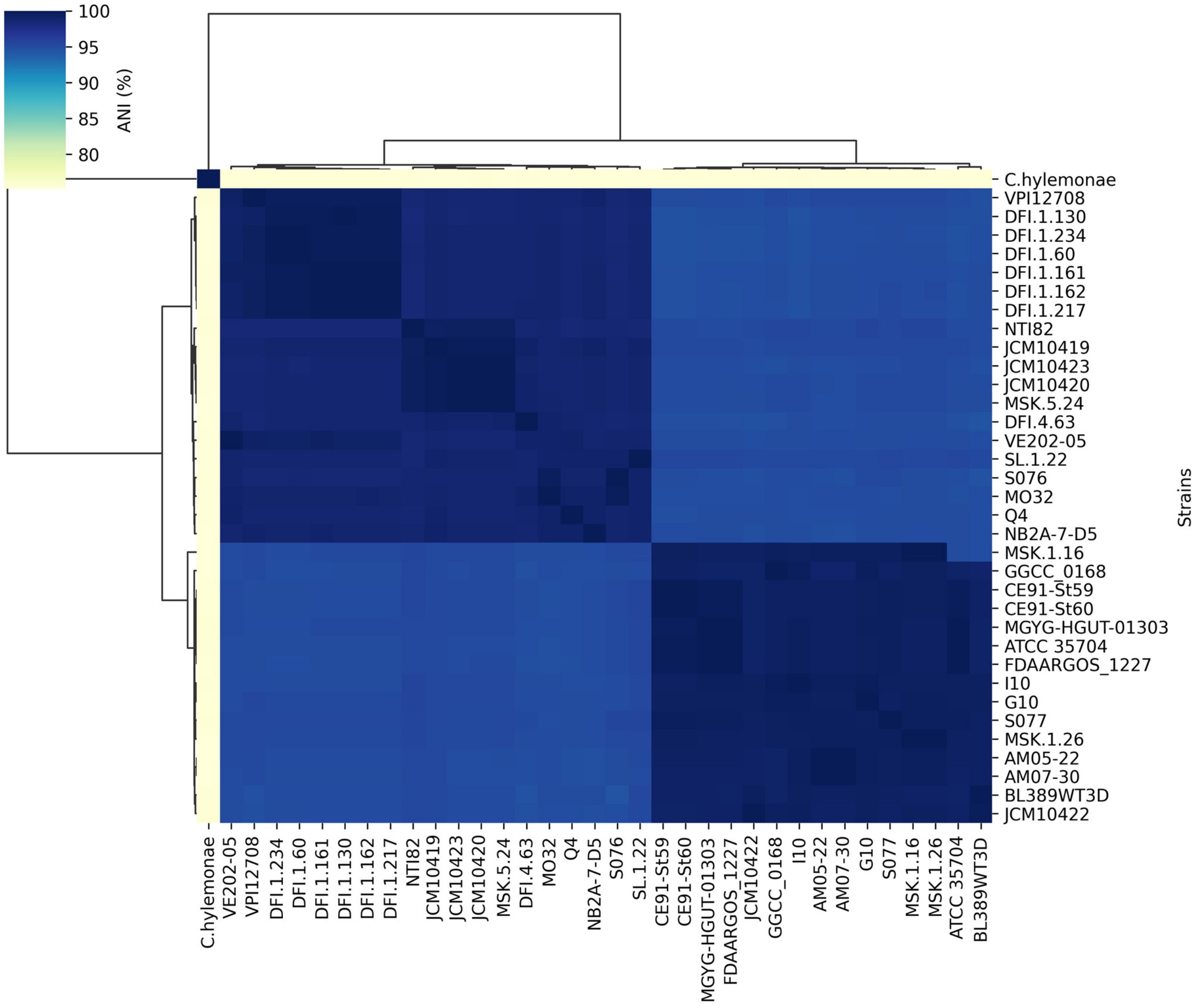

Supplementary Figure S2. ANI analysis between the *C. scindens* genomes and the *C. hylemonae* genome. The graph shows the formation of two sets of strains with a difference of around 4-5% in their genomic sequences, represented by color intensities. One group includes 15 strains and the other 19.

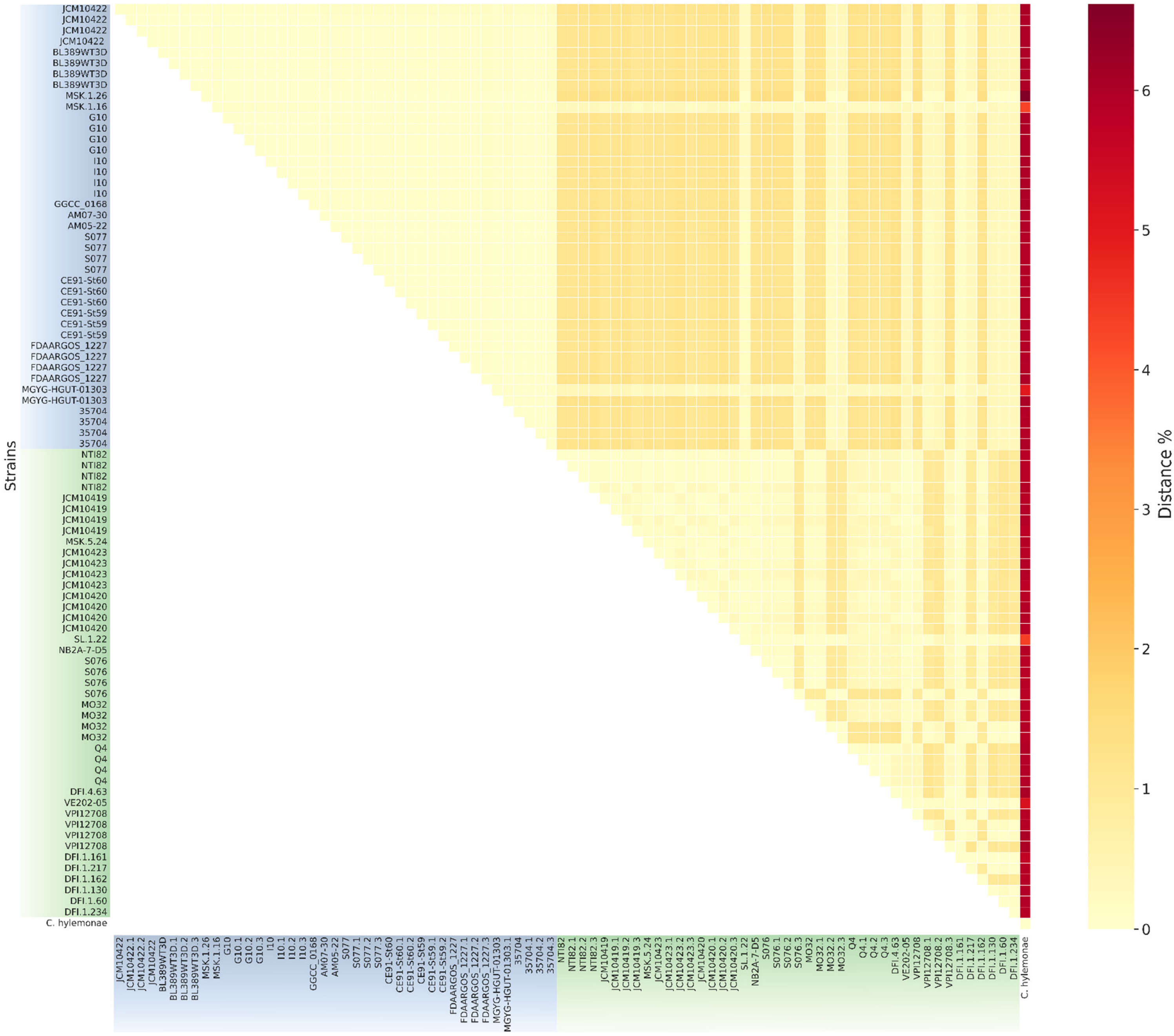

Supplementary Figure S3. Distance matrix based on the SSU rRNA gene copies of the 34 *C. scindens* strains. The species *C. hylemonae* was included as an outgroup. The distance value is shown in color; the highest value (6) in red and the lowest (0) in yellow. Strains from groups 1 and 2 are indicated by blue and green boxes respectively. The numbers used to color the matrix are presented in **Supplementary File 2**.

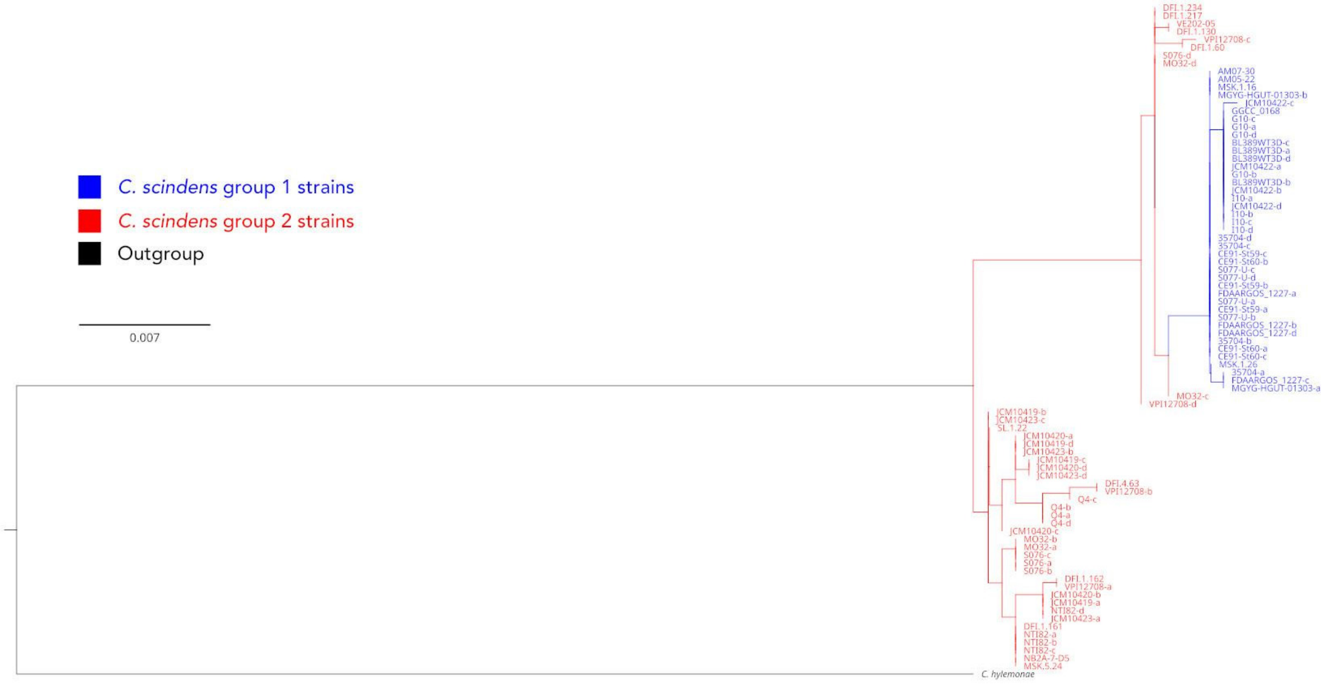

Supplementary Figure S4. Maximum likelihood phylogenetic tree of all SSU rRNA genes identified from 34 *C. scindens* genomes, with *C. hylemonae* as the outgroup. Bootstrap values were all below 50 and are therefore not shown. Strain grouping indicated by colors, with group 1 strains in blue and group 2 strains in red.

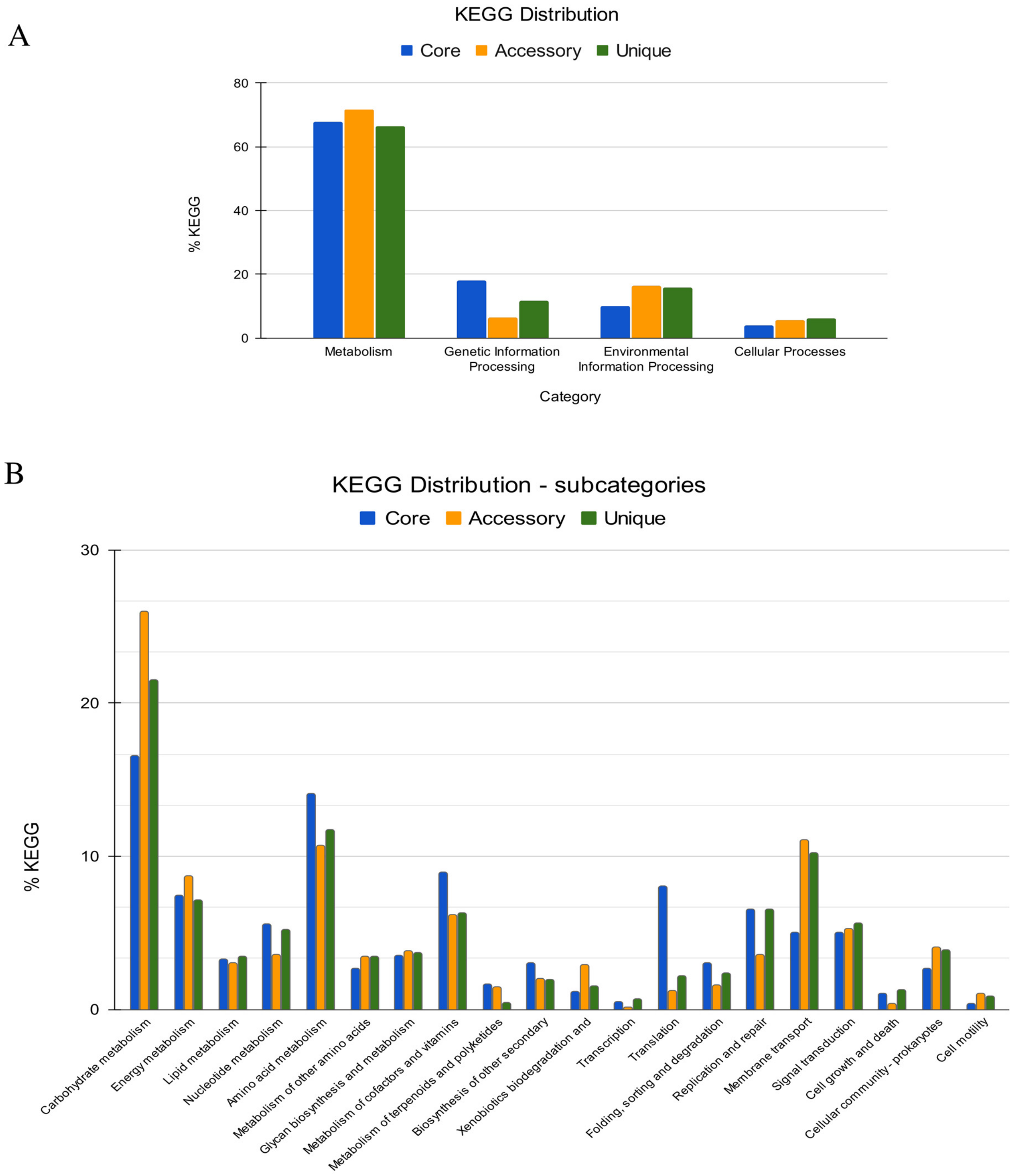

Supplementary Figure S5. KEGG distribution of core, accessory and unique genes of the *C. scindens* pangenome. **A.** Distribution in main categories. **B.** Distribution into subcategories.

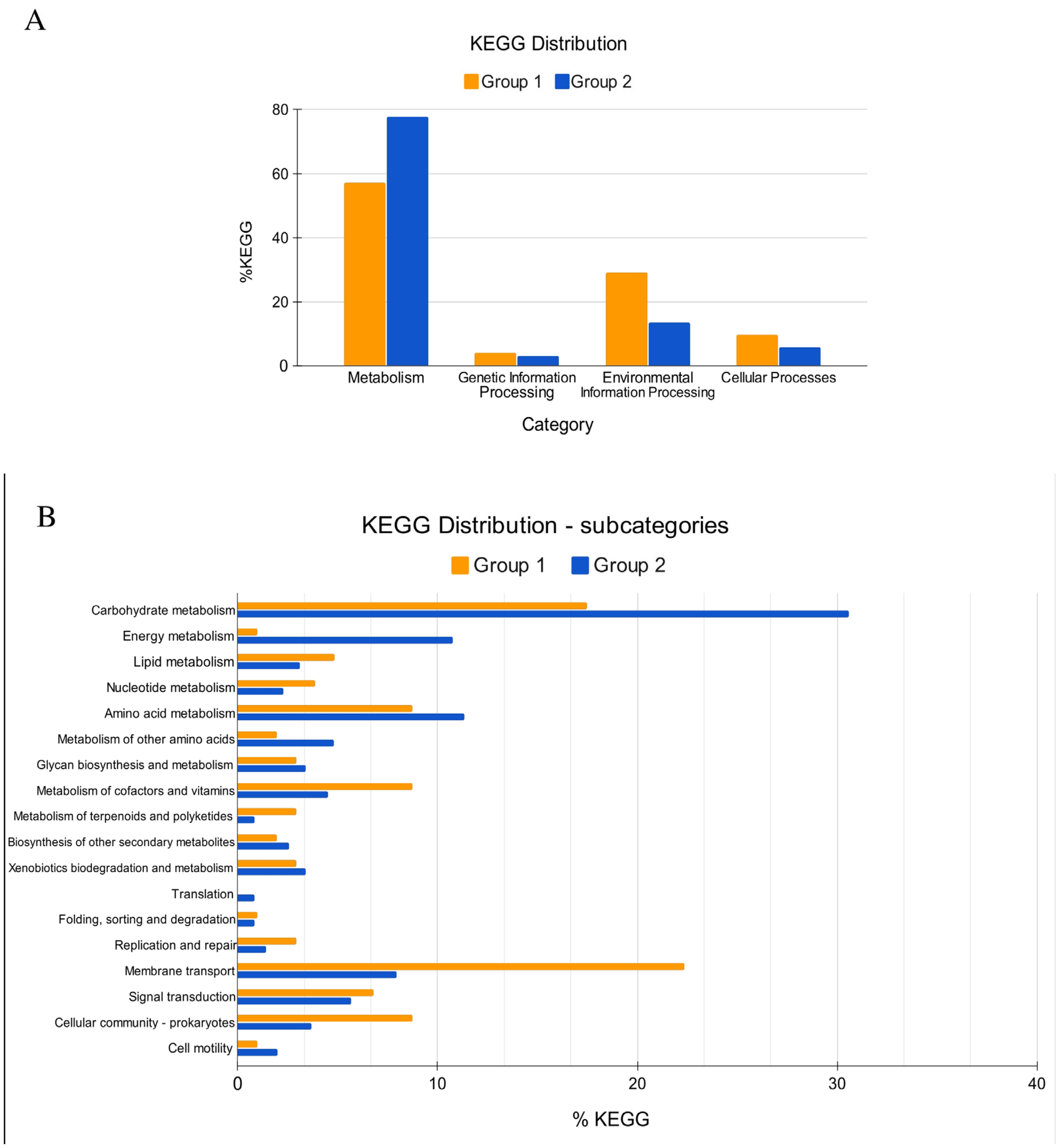

Supplementary Figure S6. KEGG distribution of the groups of *C. scindens* strains, Group 1 and Group 2. **A.** Distribution in main categories. **B.** Distribution into subcategories.

Supplementary table S1: Accession numbers for all reference genes used in the Bai and Des protein searches

Reference genes	Accession number
baiA_2	QBF74037.1
baiB	QBF74034.1
baiCD	QBF74035.1
baiE	QBF74036.1
baiF	QBF74038.1
baiG	QBF74039.1
baiH	QBF74040.1
baiI	QBF74041.1
baiJ	ACF20978.1
baiK	ACF20979.1
baiN	QBF75607.1
baiP	QBF76035.1
desA	QBF73467.1
desB	QBF73468.1
desC	QBF73469.1

Supplementary table S2: CheckM results for 34 cultivated *C. scindens* strains, including the genomes recently published by our group and other complete and incomplete genomes from NCBI.

Strain	Number of markers	Number of marker sets	Markers with 0 copies	Markers with 1 copy	Markers with 2 copies	Completeness %	Contamination %
ATCC 35704	333	171	1	330	2	99.42	1.17
AM05-22	333	171	1	332	0	99.42	0
AM07-30	333	171	1	332	0	99.42	0
BL389WT3D	333	171	1	331	1	99.42	0.58
CE91-St59	333	171	1	332	0	99.42	0
CE91-St60	333	171	1	332	0	99.42	0
DFI.1.130	333	171	1	320	12	99.42	2.46
DFI.1.161	333	171	2	330	1	98.83	0.19

DFI.1.162	333	171	1	332	0	99.42	0
DFI.1.217	333	171	1	332	0	99.42	0
DFI.1.234	333	171	2	329	2	98.83	0.29
DFI.1.60	333	171	2	331	0	98.83	0
DFI.4.63	333	171	2	330	1	98.83	0.29
FDAARGOS_1227	333	171	1	332	0	99.42	0
G10	333	171	3	330	0	98.64	0
GGCC_0168	333	171	1	327	5	99.42	1.27
I10	333	171	1	332	0	99.42	0
JCM10419	333	171	2	331	0	98.83	0
JCM10420	333	171	2	331	0	98.83	0
JCM10422	333	171	1	332	0	99.42	0
JCM10423	333	171	1	332	0	99.42	0
MGYG-HGUT-01303	333	171	1	332	0	99.42	0
MO32	333	171	2	331	0	98.83	0
MSK.1.16	333	171	1	332	0	99.42	0
MSK.1.26	333	171	1	332	0	99.42	0
MSK.5.24	333	171	2	331	0	98.83	0
NB2A-7-D5	333	171	2	331	0	98.83	0
NTI82	333	171	2	331	0	98.83	0
Q4	333	171	2	331	0	98.83	0
S076	333	171	2	331	0	98.83	0
S077	333	171	1	332	0	99.42	0
SL.1.22	333	171	2	331	0	98.83	0
VE202-05	333	171	15	317	1	94.13	0.29
VPI12708	333	171	2	331	0	98.83	0

Supplementary table S3. Genomic characteristics of 200 non-dereplicated *C. scindens* MAGs. Genome completeness values are shown in descending order.

Genome ID	Completeness %	Contamination %	Genome size (bp)	N° scaffolds	N° contigs	N50 (scaffolds)	N50 (contigs)	N° predicted genes
GCA_945830785.1_SRR5240736_bin.6_metaWRAP_v1.3_MAG_genomic	99.42	0.00	3,099,469	82	82	87,848	87,848	3,060
GCA_945875235.1_ERR1855542_bin.22_metaWRAP_v1.3_MAG_genomic	99.42	0.00	3,127,405	84	84	87,848	87,848	3,086
MGYG000001303	99.42	0.00	3,622,605	41	68	158,586	157,623	3,602
MGYG000176389	99.42	0.00	3,619,905	68	68	157,623	157,623	3,603
CokerMO_2019_SRR8692192_bin.27	99.42	0.00	2,981,811	74	74	68,939	68,939	2,913
MGYG000253462	99.22	0.00	3,336,973	64	64	107,968	107,968	3,374
MGYG000091745	99.22	0.58	3,339,557	51	64	165,625	107,968	3,364
YuJ_2015__SZAXPI003422-11__bin.45	98.83	0.00	3,780,218	44	44	140,415	140,415	3,568
MGYG000137000	98.83	0.19	3,780,218	44	44	140,415	140,415	3,568
CasaburiG_2019_SRR6277114_bin.5	98.83	0.19	3,885,654	21	21	251,049	251,049	3,672
MGYG000125324	98.83	0.00	4,056,916	17	25	546,338	291,830	3,836

CokerMO_2019_SRR8692178_bin.1	98.83	0.19	3,795,272	22	22	290,238	290,238	3,602
MGYG000229429	98.83	0.00	3,131,882	57	57	159,529	159,529	3,133
CokerMO_2019_SRR8692210_bin.15	98.83	0.00	2,953,419	70	70	82,650	82,650	2,886
MGYG000142659	98.80	0.00	3,676,932	23	23	249,655	249,655	3,426
MGYG000225303	98.71	0.00	4,059,414	20	31	545,924	265,229	3,838
HeQ_2017__RSZAXPI003099-133__bin.1	98.45	0.00	3,090,863	48	48	138,734	138,734	3,042
MGYG000209074	98.45	0.00	3,090,863	48	48	138,734	138,734	3,042
GCA_905206435.1_ERR1600561-mag-bin.52_genomic	98.25	0.88	2,985,519	63	63	85,679	85,679	2,985
MGYG000004963	98.25	0.88	2,985,519	63	63	85,679	85,679	2,985
IjazUZ_2017__S14_a_WGS__bin.19	98.11	0.12	3,562,888	211	211	23,628	23,628	3,489
MGYG000078756	98.11	0.12	3,562,888	211	211	23,628	23,628	3,489
GCA_945871535.1_SRR17382097_bin.48_metaWRAP_v1.3_MAG_genomic	97.66	0.58	2,878,239	103	103	50,587	50,587	2,896
LomanNJ_2013__OBK1196__bin.13	97.40	0.58	2,608,426	185	185	21,622	21,622	2,621
MGYG000260488	97.40	0.00	2,608,426	185	185	21,622	21,622	2,621
MGYG000009362	97.37	0.00	3,915,227	26	26	297,304	297,304	3,882
MurphyR_2019_SRR7351869_bin.8	97.08	0.00	3,771,567	53	53	112,091	112,091	3,608
GCA_945908315.1_ERR1606358_bin.2_metaWRAP_v1.3_MAG_genomic	97.08	0.00	2,946,002	57	57	93,160	93,160	2,902
Baumann-DudenhoefferAM_2018_SRR7217830_bin.1	97.08	0.00	2,890,901	68	68	79,872	79,872	2,873
YuJ_2015__SZAXPI017595-169__bin.30	97.05	0.00	3,673,145	34	34	176,479	176,479	3,423
MGYG000176351	97.05	0.58	3,673,145	34	34	176,479	176,479	3,423
YuJ_2015__SZAXPI003428-6__bin.7	96.88	0.00	3,586,629	151	151	35,075	35,075	3,455
MGYG000081734	96.88	0.00	3,586,629	151	151	35,075	35,075	3,455
YuJ_2015__SZAXPI003415-12__bin.8	96.72	0.22	3,591,634	250	250	24,910	24,910	3,589
MGYG000234009	96.72	0.22	3,591,634	250	250	24,910	24,910	3,589
MGYG000274932	96.72	2.24	3,657,773	35	35	160,445	160,445	3,443
HeQ_2017__RSZAXPI003080-114__bin.30	96.67	2.24	2,763,047	80	80	55,843	55,843	2,747
MGYG000238973	96.67	0.00	2,763,047	80	80	55,843	55,843	2,747
LiuW_2016__SRR3992985__bin.73	96.11	0.39	2,560,880	200	200	17,515	17,515	2,523
MGYG000067279	96.11	0.39	2,560,880	200	200	17,515	17,515	2,523
MurphyR_2019_SRR7352056_bin.5	96.11	1.95	3,554,900	152	152	40,200	40,200	3,401
MGYG000233132	96.01	1.95	2,960,856	79	79	66,313	66,313	2,976
GeversD_2014__SKBSTL008__bin.83	96.00	0.97	3,536,107	182	182	26,649	26,649	3,421
MGYG000228970	96.00	1.75	3,536,107	182	182	26,649	26,649	3,421
MGYG000246300	95.81	0.00	2,569,623	357	357	10,250	10,250	2,772
MGYG000212934	95.76	0.00	3,362,959	282	282	16,032	16,032	3,379
HeQ_2017__SZAXPI029501-104__bin.36	95.75	1.85	3,082,792	43	43	152,185	152,185	3,056
MGYG000179895	95.75	0.99	3,082,792	43	43	152,185	152,185	3,056
MGYG000215940	95.42	0.00	3,594,021	92	92	62,308	62,308	3,379
MGYG000288571	95.32	0.00	3,465,768	170	170	29,462	29,462	3,361
GCA_022777065.1_ASM2277706v1_genomic	94.93	1.56	2,827,450	33	33	151,255	151,255	2,810
MGYG000239782	94.86	0.12	3,590,894	82	82	89,011	89,011	3,412
GCA_004558675.1_ASM455867v1_genomic	94.74	0.88	2,885,217	99	99	48,377	48,377	2,929
FengQ_2015__SID530450__bin.66	94.74	0.29	3,296,439	44	44	147,477	147,477	3,090
MGYG000108396	94.74	0.29	3,296,439	44	44	147,477	147,477	3,090
MGYG000237452	94.46	4.18	2,760,783	82	82	53,616	53,616	2,732
QinJ_2012__DLF012__bin.15	94.45	0.29	2,523,043	260	260	14,037	14,037	2,645
MGYG000230541	94.45	0.29	2,523,043	260	260	14,037	14,037	2,645

MGYG000173046	94.15	0.00	3,464,751	38	38	164,863	164,863	3,228
MGYG000027307	94.13	0.29	3,912,387	102	102	72,302	72,302	4,593
MGYG000250872	93.97	1.07	2,813,650	49	49	92,921	92,921	2,770
MGYG000049012	93.70	0.44	3,426,874	286	286	18,132	18,132	3,408
MGYG000027911	93.65	2.19	3,456,019	320	320	14,732	14,732	3,256
MGYG000117810	93.54	0.00	2,698,782	35	35	132,487	132,487	2,622
LoombaR_2017__SID1048_bav__bin.25	93.54	0.00	2,746,452	37	37	132,487	132,487	2,661
MGYG000200365	93.54	0.00	2,746,452	37	37	132,487	132,487	2,661
YuJ_2015__SZAXPI003424-12__bin.59	93.45	0.00	3,453,537	251	251	21,299	21,299	3,393
MGYG000204864	93.45	0.00	3,453,537	251	251	21,299	21,299	3,393
MGYG000115572	93.28	0.29	2,494,484	244	244	14,183	14,183	2,605
FengQ_2015__SID531403__bin.12	93.04	0.58	2,718,000	270	270	14,354	14,354	2,902
MGYG000175564	93.04	0.58	2,718,000	270	270	14,354	14,354	2,902
MGYG000054548	92.90	1.42	3,104,168	528	528	8,293	8,293	3,332
YuJ_2015__SZAXPI015233-19__bin.6	92.89	0.08	3,493,459	72	72	71,570	71,570	3,271
MGYG000240459	92.89	0.08	3,493,459	72	72	71,570	71,570	3,271
MGYG000190421	92.85	0.88	2,453,338	334	334	10,302	10,302	2,639
LoombaR_2017__SID5639_uuc__bin.9	92.79	0.97	3,618,456	166	166	31,893	31,893	3,550
MGYG000092190	92.79	0.97	3,618,456	166	166	31,893	31,893	3,550
ZellerG_2014__CCIS88007743ST-4-0__bin.21	92.69	0.70	3,379,725	146	146	36,321	36,321	3,257
MGYG000243613	92.69	0.70	3,379,725	146	146	36,321	36,321	3,257
NielsenHB_2014__V1_UC11_5__bin.53	92.67	0.00	2,786,591	116	116	37,909	37,909	2,838
MGYG000179685	92.67	0.00	2,786,591	116	116	37,909	37,909	2,838
QinJ_2012__T2D-016__bin.41	92.23	0.88	3,334,283	447	447	9,994	9,994	3,473
MGYG000278088	92.23	0.88	3,334,283	447	447	9,994	9,994	3,473
MGYG000232691	91.93	1.46	3,524,914	100	100	5,6167	56,167	3,339
NielsenHB_2014__V1_CD7_4__bin.41	91.81	0.00	2,729,357	47	47	110,798	110,798	2,685
MGYG000093362	91.81	0.00	2,729,357	47	47	110,798	110,798	2,685
HeQ_2017__SZAXPI029463-136__bin.44	91.40	0.58	3,490,822	47	47	111,242	111,242	3,270
MGYG000038545	91.40	0.58	3,490,822	47	47	111,242	111,242	3,270
IjazUZ_2017__S102_a_WGS__bin.2	90.74	2.52	2,389,371	323	323	10,060	10,060	2,601
MGYG000191948	90.74	2.52	2,389,371	323	323	10,060	10,060	2,601
HeQ_2017__SZAXPI029483-78__bin.3	90.60	0.16	3,375,410	285	285	17,693	17,693	3,371
MGYG000126456	90.60	0.16	3,375,410	285	285	17,693	17,693	3,371
MGYG000025974	90.35	1.95	3,549,982	45	45	118,974	118,974	3,291
BackhedF_2015_ERR526080_bin.17	90.31	3.22	2,454,954	549	549	5,756	5,756	2,850
IjazUZ_2017__S47_a_WGS__bin.1	90.07	1.66	2,468,661	376	376	8,813	8,813	2,778
MGYG000084316	90.07	1.66	2,468,661	376	376	8,813	8,813	2,778
XieH_2016__YSZC12003_37190R1__bin.30	89.93	0.23	3,463,597	295	295	17,602	17,602	3,585
MGYG000089331	89.93	0.23	3,463,597	295	295	17,602	17,602	3,585
MurphyR_2019_SRR7351692_bin.11	89.71	0.94	3,336,402	474	474	8,878	8,878	3,442
MGYG000057334	89.60	4.19	3,406,321	315	315	15,510	15,510	3,410
GeversD_2014__SKBSTL041__bin.56	88.77	1.42	2,388,451	333	333	9,296	9,296	2,559
MGYG000104828	88.77	1.42	2,388,451	333	333	9,296	9,296	2,559
MGYG000260649	88.71	1.41	2,329,935	534	534	5,404	5,404	2,682
MGYG000183293	88.69	0.58	2,364,329	217	217	15,442	15,442	2,467
XieH_2016__YSZC12003_37179__bin.80	88.54	1.02	2,424,128	196	196	17,990	17,990	2,522
MGYG000115790	88.54	1.02	2,424,128	196	196	17,990	17,990	2,522
MGYG000033510	88.30	0	2,350,964	177	177	19,126	19,126	2,279
MGYG000067284	88.21	1.75	2,308,534	581	581	4,957	4,957	2,669

FengQ_2015__SID531333__bin.47	87.76	1.36	2,352,310	297	297	10,508	10,508	2,518
MGYG000100546	87.76	1.36	2,352,310	297	297	10,508	10,508	2,518
BackhedF_2015__SID87_12M__bin.39	87.52	1.17	2,414,812	229	229	15,282	15,282	2,533
MGYG000154256	87.52	1.17	2,414,812	229	229	15,282	15,282	2,533
MGYG000197807	87.49	4.33	3,325,017	278	278	17,210	17,210	3,287
HeQ_2017__SZAXPI029564-74__bin.70	87.45	0.49	3,481,952	348	348	14,806	14,806	3,573
MGYG000126162	87.45	0.49	3,481,952	348	348	14,806	14,806	3,573
MGYG000234569	87.23	1.02	3,405,590	268	268	19,246	19,246	3,385
MGYG000014135	86.86	1.85	3,231,296	415	415	10,502	10,502	3,079
MGYG000275853	86.83	0.15	3,393,119	147	147	38,385	38,385	3,258
MGYG000201716	86.07	3.31	2,327,850	285	285	10,587	10,587	2,455
BackhedF_2015__SID39_12M__bin.41	86.03	1.57	2,357,953	404	404	7,186	7,186	2,660
MGYG000259622	86.03	1.57	2,357,953	404	404	7,186	7,186	2,660
MGYG000147437	85.96	1.07	3,298,593	335	335	13,445	13,445	3,363
FengQ_2015__SID31367__bin.39	85.59	2.24	3,330,554	434	434	10,985	10,985	3,417
MGYG000115658	85.59	2.24	3,330,554	434	434	10,985	10,985	3,417
MGYG000108964	85.01	1.17	2,446,854	241	241	12,275	12,275	2,604
RaymondF_2016__P20E7__bin.20	84.73	0.19	2,229,022	351	351	7,954	7,954	2,237
MGYG000239495	84.73	0.19	2,229,022	351	351	7,954	7,954	2,237
MGYG000214544	84.13	2.25	2,648,863	44	44	88,733	88,733	2,612
MGYG000237576	83.90	1.07	2,949,887	794	794	4,354	4,354	3,351
MGYG000032531	83.33	0	2,441,556	69	69	53,698	53,698	2,298
MGYG000183297	83.18	0.90	3,014,150	734	734	5,108	5,108	3,337
BackhedF_2015_ERR525896_bin.18	81.85	2.12	2,115,841	709	709	3,558	3,558	2,630
MGYG000049970	81.20	0.90	2,394,241	342	342	8,462	8,462	2,655
MGYG000217487	81.09	2.11	2,180,542	669	669	3,949	3,949	2,655
IjazUZ_2017__S149_a_WGS__bin.14	79.80	0.98	3,141,993	724	724	5,018	5,018	3,716
MGYG000180135	79.80	0.98	3,141,993	724	724	5,018	5,018	3,716
MGYG000165712	79.74	2.89	2,076,098	699	699	3,469	3,469	2,549
FengQ_2015__SID31537__bin.55	78.98	0.36	2,147,458	415	415	6,408	6,408	2,428
MGYG000089002	78.98	0.36	2,147,458	415	415	6,408	6,408	2,428
MGYG000276115	78.89	3.49	2,185,273	355	355	7,365	7,365	2,435
MGYG000146602	78.63	0.82	3,156,989	261	261	18,098	18,098	3,130
MGYG000151937	78.37	1.17	1,981,994	726	726	3,156	3,156	2,561
MGYG000082223	77.99	0.88	3,189,959	58	58	90,158	90,158	2,995
IjazUZ_2017__S46_a_WGS__bin.2	77.88	2.90	2,083,701	554	554	4,255	4,255	2,634
MGYG000100111	77.88	2.90	2,083,701	554	554	4,255	4,255	2,634
BackhedF_2015_ERR525961_bin.14	77.75	1.27	1,913,827	845	845	2,498	2,498	2,598
ZellerG_2014__CCIS24254057ST-4-0__bin.15	77.75	1.27	2,953,898	637	637	5,438	5,438	2,999
MGYG000141644	77.75	3.57	2,953,898	637	637	5,438	5,438	2,999
CokerMO_2019_SRR8692181_bin.17	77.46	0.88	2,790,896	1072	1072	3,024	3,024	3,529
GeversD_2014__SKBSTL016__bin.45	76.81	0.94	1,960,615	525	525	4,158	4,158	2,312
MGYG000078202	76.81	0.94	1,960,615	525	525	4,158	4,158	2,312
MurphyR_2019_SRR7411324_bin.13	76.35	2.26	2,990,792	768	768	4,599	4,599	3,408
IjazUZ_2017__S15_a_WGS__bin.1	75.57	3.12	2,843,086	778	778	3,986	3,986	3,403
MGYG000257293	75.57	3.12	2,843,086	778	778	3,986	3,986	3,403
MGYG000127397	75.04	0.68	2,948,052	429	429	7,895	7,895	3,086
MGYG000177750	74.91	0.00	1,923,449	318	318	7,068	7,068	2,124
MGYG000235414	74.61	3.02	2,032,034	670	670	3,349	3,349	2,500
LiJ_2014__V1.CD54-0__bin.3	74.48	3.45	3,487,032	334	334	18,927	18,927	3,503
MGYG000032252	74.48	3.45	3,487,032	334	334	18,927	18,927	3,503

FengQ_2015__SID31137__bin.48	73.47	1.58	1,918,844	512	512	4,138	4,138	2,263
MGYG000196552	73.47	1.58	1,918,844	512	512	4,138	4,138	2,263
BackhedF_2015__SID546_4M__bin.12	73.33	1.46	2,045,906	510	510	4,738	4,738	2,421
MGYG000156646	73.33	1.46	2,045,906	510	510	4,738	4,738	2,421
FengQ_2015__SID31883__bin.30	72.82	2.40	2,849,860	688	688	4,633	4,633	3,372
MGYG000059009	72.82	2.40	2,849,860	688	688	4,633	4,633	3,372
MGYG000161697	72.24	1.07	2,572,678	527	527	5,408	5,408	2,880
MGYG000230250	70.77	3.61	2,099,501	302	302	9,043	9,043	2,285
MurphyR_2019_SRR7351790_bin.1	70.29	2.24	2,556,966	732	732	3,943	3,943	2,904
LiJ_2014__V1.CD3-0-PN__bin.36	70.14	1.72	2,258,476	356	356	8,325	8,325	2,523
MGYG000230595	70.14	1.72	2,258,476	356	356	8,325	8,325	2,523
MGYG000043161	69.20	0.74	2,568,057	1036	1036	2,833	2,833	3,248
IjazUZ_2017__S56_a_WGS__bin.7	68.37	1.38	1,871,279	535	535	3,845	3,845	2,395
MGYG000260044	68.37	1.38	1,871,279	535	535	3,845	3,845	2,395
MGYG000189753	68.02	0.58	1,888,580	416	416	5,171	5,171	2,204
MGYG000288033	67.90	2.59	2,468,913	539	539	5,087	5,087	2,774
BackhedF_2015__SID546_12M__bin.24	67.48	1.27	1,807,772	508	508	3,782	3,782	2,211
MGYG000118803	67.48	1.27	1,807,772	508	508	3,782	3,782	2,211
MGYG000158936	66.28	1.07	1,657,119	330	330	5,447	5,447	1,871
NielsenHB_2014__V1_UC11_0__bin.24	65.91	1.88	2,035,703	466	466	5,282	5,282	2,414
MGYG000027702	65.91	1.88	2,035,703	466	466	5,282	5,282	2,414
MGYG000239731	65.50	2.59	2,745,202	501	501	6,282	6,282	2,970
MGYG000065110	65.10	0.25	2,152,440	46	46	76,086	76,086	2,143
YuJ_2015__SZAXPI015211-166__bin.3	64.53	1.17	2,703,826	720	720	4,254	4,254	3,101
MGYG000212469	64.53	1.17	2,703,826	720	720	4,254	4,254	3,101
MGYG000256913	62.28	1.23	2,683,607	526	526	5,439	5,439	2,930
MGYG000019508	61.92	0.58	1,489,172	681	681	2,371	2,371	1,926
MGYG000286990	61.80	0.12	2,356,565	442	442	5,838	5,838	2,473
YuJ_2015__SZAXPI017457-24__bin.57	60.42	1.72	2,389,318	762	762	3,283	3,283	2,900
MGYG000060898	60.42	1.72	2,389,318	762	762	3,283	3,283	2,900
ParnanenK_2018_SRR5723857_bin.3	59.51	0.88	1,513,562	752	752	2,147	2,147	1,988
IjazUZ_2017__S16_a_WGS__bin.12	58.33	2.24	2,275,143	806	806	2,886	2,886	3,013
MGYG000277528	58.16	1.27	1,584,323	392	392	4,166	4,166	1,894
BackhedF_2015__SID577_12M__bin.28	56.30	1.72	1,660,682	495	495	3,684	3,684	2,055
XieH_2016__YSZC12003_37400__bin.34	55.99	1.75	2,235,680	647	647	3,753	3,753	2,709
NielsenHB_2014__V1_UC16_0__bin.1	55.52	0.00	2,263,928	684	684	3,521	3,521	2,731
MGYG000245088	55.52	0.00	2,263,928	684	684	3,521	3,521	2,731
IjazUZ_2017__S48_a_WGS__bin.2	54.62	1.72	1,559,077	524	524	3,146	3,146	2,096
BackhedF_2015_ERR525992_bin.38	54.28	0.00	1,371,348	732	732	1,984	1,984	1,911
VincentC_2016__MM063__bin.1	50.78	0.00	1,583,257	561	561	2,874	2,874	2,021
MGYG000107506	50.78	0.00	1,583,257	561	561	2,874	2,874	2,021

Supplementary table S4: Genomic characteristics of 58 dereplicated MAGs. Genome completeness values are shown in descending order.

Genome ID	Completeness %	Contamination %	Genome size (bp)	N° scaffolds	N° contigs	N50 (scaffolds)	N50 (contigs)	N° predicted genes
CokerMO_2019_SRR8692192_bin.27	99.42	0.00	2,981,811	74	74	68,939	68,939	2.913
GCA_009696415.1_ASM969641v1_genomic	99,42	0.00	3,614,384	114	114	87,879	87,879	3.523
CasaburiG_2019_SRR6277114_bin.5	98.83	0.19	3,885,654	21	21	251,049	251,049	3.672
MGYG000125324	98.83	0.00	4,056,916	17	25	546,338	291,830	3.836
HeQ_2017__RSZAXPI003099-133__bin.1	98.45	0.00	3,090,863	48	48	138,734	138,734	3.042
IjazUZ_2017__S14_a_WGS__bin.19	98.11	0.12	3,562,888	211	211	23,628	23,628	3.489
MGYG000246300	95.81	0.00	2,569,623	357	357	10,250	10,250	2.772
MGYG000212934	95.76	0.00	3,362,959	282	282	16,032	16,032	3.379
GCA_945830785.1_SRR5240736_bin.6_metaWRAP_v1.3_MAG_genomic	99.42	0.00	3,099,469	82	82	87,848	87,848	3.060
GCA_945908315.1_ERR1606358_bin.2_metaWRAP_v1.3_MAG_genomic	97.08	0.00	2,946,002	57	57	93,160	93,160	2.902
GCA_004558675.1_ASM455867v1_genomic	94.74	0.88	2,885,217	99	99	48,377	48,377	2.929
LoombaR_2017__SID5639_uuc__bin.9	92.79	0.97	3,618,456	166	166	31,893	31,893	3.550
IjazUZ_2017__S102_a_WGS__bin.2	90.74	2.52	2,389,371	323	323	10,060	10,060	2.601
MGYG000089331	89.93	0.23	3,463,597	295	295	17,602	17,602	3.585
MurphyR_2019_SRR7351692_bin.11	89.71	0.94	3,336,402	474	474	8,878	8,878	3.442
MGYG000260649	88.71	1.41	2,329,935	534	534	5,404	5,404	2.682
MGYG000067284	88.21	1.75	2,308,534	581	581	4,957	4,957	2.669
FengQ_2015__SID531333__bin.47	87.76	1.36	2,352,310	297	297	10,508	10,508	2.518
MGYG000201716	86.07	3.31	2,327,850	285	285	10,587	10,587	2.455
MGYG000239495	84.73	0.19	2,229,022	351	351	7,954	7,954	2.237
MGYG000032531	83.33	0	2,441,556	69	69	53,698	53,698	2.298
MGYG000049970	81.20	0.90	2,394,241	342	342	8,462	8,462	2.655
MGYG000217487	81.09	2.11	2,180,542	669	669	3,949	3,949	2.655
IjazUZ_2017__S149_a_WGS__bin.14	79.80	0.98	3,141,993	724	724	5,018	5,018	3.716
FengQ_2015__SID31537__bin.55	78.98	0.36	2,147,458	415	415	6,408	6,408	2.428
MGYG000151937	78.37	1.17	1,981,994	726	726	3,156	3,156	2.561
IjazUZ_2017__S46_a_WGS__bin.2	77.88	2.90	2,083,701	554	554	4,255	4,255	2.634
MGYG000141644	77.75	3.57	2,953,898	637	637	5,438	5,438	2.999
CokerMO_2019_SRR8692181_bin.17	77.46	0.88	2,790,896	1072	1072	3,024	3,024	3.529
GeversD_2014__SKBSTL016__bin.45	76.81	0.94	1,960,615	525	525	4,158	4,158	2.312
MurphyR_2019_SRR7411324_bin.13	76.35	2.26	2,990,792	768	768	4,599	4,599	3.408
IjazUZ_2017__S15_a_WGS__bin.1	75.57	3.12	2,843,086	778	778	3,986	3,986	3.403
MGYG000177750	74.91	0.00	1,923,449	318	318	7,068	7,068	2.124
MGYG000235414	74.61	3.02	2,032,034	670	670	3,349	3,349	2.500
FengQ_2015__SID31137__bin.48	73.47	1.58	1,918,844	512	512	4,138	4,138	2.263
BackhedF_2015__SID546_4M__bin.12	73.33	1.46	2,045,906	510	510	4,738	4,738	2.421
MGYG000161697	72.24	1.07	2,572,678	527	527	5,408	5,408	2.880
LiJ_2014__V1.CD3-0-PN__bin.36	70.14	1.72	2,258,476	356	356	8,325	8,325	2.523
MurphyR_2019_SRR7351790_bin.1	70.29	2.24	2,556,966	732	732	3,943	3,943	2.904
IjazUZ_2017__S56_a_WGS__bin.7	68.37	1.38	1,871,279	535	535	3,845	3,845	2.395
MGYG000288033	67.90	2.59	2,468,913	539	539	5,087	5,087	2.774
BackhedF_2015__SID546_12M__bin.24	67.48	1.27	1,807,772	508	508	3,782	3,782	2.211

MGYG000158936	66.28	1.07	1,657,119	330	330	5,447	5,447	1.871
MGYG000027702	65.91	1.88	2,035,703	466	466	5,282	5,282	2.414
MGYG000239731	65.50	2.59	2,745,202	501	501	6,282	6,282	2.970
MGYG000065110	65.10	0.25	2,152,440	46	46	76,086	76,086	2.143
MGYG000256913	62.28	1.23	2,683,607	526	526	5,439	5,439	2.930
MGYG000019508	61.92	0.58	1,489,172	681	681	2,371	2,371	1.926
MGYG000286990	61.80	0.12	2,356,565	442	442	5,838	5,838	2.473
MGYG000060898	60.42	1.72	2,389,318	762	762	3,283	3,283	2.900
ParnanenK_2018_SRR5723857_bin.3	59.51	0.88	1,513,562	752	752	2,147	2,147	1.988
IjazUZ_2017__S16_a_WGS__bin.12	58.33	2.24	2,275,143	806	806	2,886	2,886	3.013
BackhedF_2015__SID577_12M__bin.28	56.30	1.72	1,660,682	495	495	3,684	3,684	2.055
XieH_2016__YSZC12003_37400__bin.34	55.99	1.75	2,235,680	647	647	3,753	3,753	2.709
MGYG000245088	55.52	0.00	2,263,928	684	684	3,521	3,521	2.731
IjazUZ_2017__S48_a_WGS__bin.2	54.62	1.72	1,559,077	524	524	3,146	3,146	2.096
BackhedF_2015_ERR525992_bin.38	54.28	0.00	1,371,348	732	732	1,984	1,984	1.911
MGYG000107506	50.78	0.00	1,583,257	561	561	2,874	2,874	2.021

Supplementary File S1. Sequence alignment generated by Muscle for all SSU rRNA genes identified in 34 *C. scindens* genomes and in *C. hylemonae*.

Supplementary File S2. Uncorrected distance matrix between all SSU rRNA gene sequences identified in 34 *C. scindens* genomes and in *C. hylemonae*, calculated by distmat of the EMBOSS suite.
